# Combined Inhibition of *UBE2C* and *PLK1* Reduce Cell Proliferation and Arrest Cell Cycle by Affecting *ACLY* in Pan-Cancer

**DOI:** 10.3390/ijms242115658

**Published:** 2023-10-27

**Authors:** Keying Liang, Qian Wang, Li Qiu, Xiaocheng Gong, Zixi Chen, Haibo Zhang, Ke Ding, Yunfei Liu, Jinfen Wei, Shudai Lin, Shuying Fu, Hongli Du

**Affiliations:** School of Biology and Biological Engineering, South China University of Technology, Guangzhou 510006, China; 201910107991@mail.scut.edu.cn (K.L.); 202021049123@mail.scut.edu.cn (Q.W.); qiuli0531@gmail.com (L.Q.); 202010108425@mail.scut.edu.cn (X.G.); chenzixi2011@gmail.com (Z.C.); zhanghaibo2020@gmail.com (H.Z.); keding0302@gmail.com (K.D.); 202120149279@mail.scut.edu.cn (Y.L.); weijinfen@scut.edu.cn (J.W.); linsd@gdou.edu.cn (S.L.); celia.fushuying@gmail.com (S.F.)

**Keywords:** ubiquitin conjugating enzyme E2 C (UBE2C), polo-like kinase 1 (PLK1), baculoviral IAP repeat containing 5 (BIRC5), ATP-citrate lyase (ACLY), cell proliferation, cell cycle, de novo lipogenesis (DNL), pan-cancer

## Abstract

Various studies have shown that the cell-cycle-related regulatory proteins UBE2C, PLK1, and BIRC5 promote cell proliferation and migration in different types of cancer. However, there is a lack of in-depth and systematic research on the mechanism of these three as therapeutic targets. In this study, we found a positive correlation between the expression of *UBE2C* and *PLK1*/*BIRC5* in the Cancer Genome Atlas (TCGA) database, revealing a potential combination therapy candidate for pan-cancer. Quantitative real-time PCR (qRT-PCR), Western blotting (WB), cell phenotype detection, and RNA-seq techniques were used to evidence the effectiveness of the combination candidate. We found that combined interference of *UBE2C* with *PLK1* and *UBE2C* with *BIRC5* affected metabolic pathways by significantly downregulating the mRNA expression of *IDH1* and *ACLY*, which was related to the synthesis of acetyl-CoA. By combining the PLK1 inhibitor volasertib and the ACLY inhibitor bempedoic acid, it showed a higher synergistic inhibition of cell viability and higher synergy scores in seven cell lines, compared with those of other combination treatments. Our study reveals the potential mechanisms through which cell-cycle-related genes regulate metabolism and proposes a potential combined targeted therapy for patients with higher *PLK1* and *ACLY* expression in pan-cancer.

## 1. Introduction

Over the past decade, our understanding of the role of cell-cycle-related regulatory proteins in cancer and their potential as therapeutic targets has increased significantly. The success of cell-cycle-targeting therapies will depend on the development of specific and highly effective compounds, the identification of the unique vulnerabilities of tumor cells, and the availability of novel therapeutic modalities that target multiple components of different pathways. Combinations of several selective inhibitors may significantly improve their therapeutic response. The field of cyclin-dependent kinases and cell cycle checkpoints has witnessed a growing number of preclinical and clinical studies. These have unraveled new insights on their mechanisms, successfully translated this knowledge into the development of highly effective inhibitors, and proposed new perspectives on combination therapy strategies that yield clinical benefits for a substantial patient population [1,2]. However, the possible regulatory mechanisms of several important but undruggable cell-cycle-related proteins, such as ubiquitin-conjugating enzyme E2 C (UBE2C), in pan-cancer are still unknown.

In our previous study, we found that UBE2C was co-regulated by lysine acetyltransferase 2A (KAT2A) and E2F transcription factor 1 (E2F1) to promote cell proliferation and migration in pan-cancer [3]. Furthermore, a protein–protein interaction analysis indicated that UBE2C could form an interaction network with polo-like kinase 1 (PLK1) and baculoviral IAP repeat containing 5 (BIRC5, as known as survivin). Nevertheless, the co-regulation mechanism of these three genes has not been revealed. Ubiquitination-dependent proteolysis is related to various cellular processes, including cell cycle progression, signal transduction, and differentiation [4]. UBE2C is a member of the E2 ubiquitin-conjugating enzyme family [5], as an exclusive partner of APC/C, which participates in the degradation of a family of APC/C target proteins by initiating Lys11-linked ubiquitin chains [6]. Many studies have shown that the expression of UBE2C is upregulated and associated with poor clinical outcomes in a variety of human malignancies [7,8,9]. In addition to its involvement in regulating the cell cycle, multiple in vitro cell culture studies have proposed that UBE2C exerts inhibitory effects on autophagy and facilitates the proliferation of lung cancer cells through distinct signaling pathways [7,10,11]. Furthermore, several studies have demonstrated a correlation between UBE2C and the fate of p53, as it enhances p53 ubiquitination and promotes the progression of hepatocellular carcinoma and endometrial cancer [12,13]. All these findings suggest that UBE2C is closely associated with the development of cancer and could be a potential therapeutic target for different types of cancers. However, inhibitors specifically targeting UBE2C remain undiscovered, but there is evidence that a potential target for the proteasome inhibitor bortezomib may be UBE2C [14].

PLK1 is a key regulator of mitosis in eukaryotic cells. Given its prominent role in cell cycle progression, the overexpression of PLK1 is considered a cancer driver by overriding cellular checkpoints and inducing genetic instability [15]. Many studies have shown that patients with high PLK1 expression in their tumors exhibit a poorer survival rate than those with low PLK1 expression [16,17,18,19]. Nevertheless, novel substrates and functions of PLK1 continue to be unveiled in the regulation of cell cycle progression and beyond. Multiple studies have elucidated the involvement of PLK1 in governing autophagy and the DNA damage response within cancer cells [20,21,22,23], rendering it a compelling therapeutic target for diverse malignancies. Both the ATP pocket at the catalytic domain of PLK1 and the phosphopeptide-binding pocket at the PBD are being investigated as target sites for the development of novel antitumor drugs [24]. To date, at least 10 PLK1 inhibitors have been entered into clinical trials, among which volasertib (BI6727), an ATP-competitive kinase inhibitor from the dihydropteridinone class of compounds [25], can induce the formation of monopolar spindles as well as a distinct prometaphase arrest phenotype (polo-arrest) and subsequent apoptosis [26]. The binding of volasertib to the hinge region between the NH2-terminal end and COOH-terminal lobe of the kinase domain is facilitated by two hydrogen bonds. This positioning places volasertib within the ATP-binding pocket of PLK1, resulting in its catalytic inactivation. Numerous preclinical trials have consistently demonstrated that volasertib exhibits a high efficacy across a diverse range of carcinomas, thereby augmenting antitumor activity [27,28]. Notably, the FDA granted a fast track designation to volasertib in 2014 for the treatment of acute myeloid leukemia (AML) [27,29].

BIRC5 is a member of the inhibitor of apoptosis (IAP) gene family that was discovered in 1997, which encodes negative regulatory proteins that prevent apoptotic cell death [30]. The primary clinical interest in BIRC5 is as a targeted cancer therapy, as it is the fourth most upregulated mRNA in the human cancer transcriptome [31]. The disturbance of *BIRC5* expression is mainly due to transcriptional inhibition, which leads to continuous synthesis [32] and/or splicing changes throughout the cell cycle [33]. Furthermore, studies have demonstrated that the ubiquitination and deubiquitylation of the BIRC5 protein can be regulated through diverse mechanisms to modulate tumor progression [34,35]. In this context, the most studied BIRC5 inhibitor is YM155. YM155 can significantly inhibit the expression of BIRC5 at the protein and mRNA levels with high selectivity [36]. However, the further clinical development of YM155 has been less successful, and most combination regimens have failed to achieve their intended goals in various cancers, including advanced melanoma [37], HER2-negative metastatic breast cancer [38], prostate cancer [39], and non-small cell lung cancer [40].

Combination treatment strategies represent a potentially significant approach to exploiting specific metabolic vulnerabilities in particular cancer types, as well as enhancing conventional therapies and targeting cancer-specific metabolic adaptations resulting from such treatments. Therefore, we aim to explore the mechanism underlying the optimal combination of inhibitory agents for pan-tumor regulation and validate its anti-tumor efficacy. In this study, we utilized RNA-seq technology to investigate the molecular mechanism underlying the combined inhibition of the cell-cycle-related genes *UBE2C* and *PLK1*. By integrating the results from our RNA-seq analysis of the combined inhibition involving *UBE2C* and *BIRC5*, we identified two promising candidate targets, *IDH1* and *ACLY*. Our findings demonstrate that the pharmacological co-inhibition of PLK1 and ACLY significantly suppresses pan-cancer cell proliferation compared to single-agent treatment and that this combination downregulated UBE2C mRNA and protein expression, which revealed a novel potential molecular mechanism. We envision that utilizing novel combination strategies of approved inhibitors to increase their therapeutic value and minimize redundancy may provide some new insights and theoretical references for the selection of effective combination targets for cancer therapy. 

## 2. Results

### 2.1. The High Expression of UBE2C and PLK1 Could Promote Cancer Progress in Pan-Cancer

**Figure 1:**
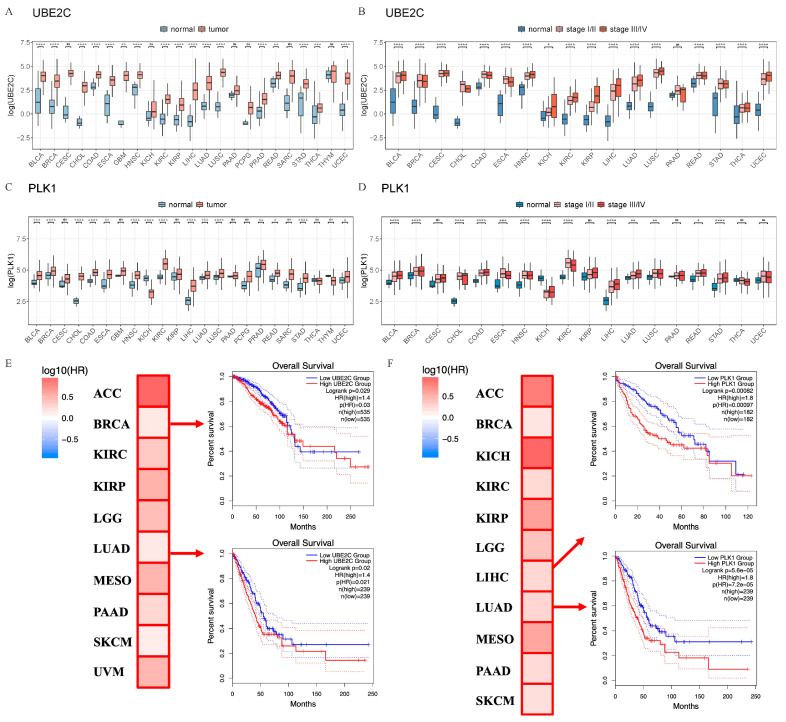
UBE2C and PLK1 are significantly upregulated in pan-cancer tissues and cells. The expression levels of UBE2C (**A**) and PLK1 (**C**) in normal tissues and tumor samples. Gene expression variance between different groups was determined using a Wilcoxon test. The expression levels of UBE2C (**B**) and PLK1 (**D**) in pathological stages. One-way ANOVA analysis with Bonferroni post hoc test. Kaplane–Meier estimates of overall survival (OS) are shown according to the expression level of UBE2C (**E**) and PLK1 (**F**) with the 95% confidence interval shown as dashed lines. Note that the patients with low groups (in blue) display better survival than those with high groups (in red). ACC, Adrenocortical carcinoma; BRCA, Breast invasive carcinoma; KICH, Kidney chromophobe; KIRC, Kidney renal clear cell carcinoma; KIRP, Kidney renal papillary cell carcinoma; LGG, Brain lower-grade glioma; LIHC, Liver hepatocellular carcinoma; LUAD, Lung adenocarcinoma; MESO, Mesothelioma; PAAD, Pancreatic adenocarcinoma; SKCM, Skin cutaneous melanoma; UVM, Uveal melanoma ns—No significance; *: *p* < 0.05; **: *p* < 0.01; ***: *p* < 0.001; ****: *p* < 0.0001.

To explore the expression of *UBE2C* and *PLK1* in pan-cancer, bioinformatics analyses were performed using the GEPIA 2.0 online software [41] and TCGA database. Compared with normal tissues, the expression level of *PLK1* was slightly higher, while that of *UBE2C* was obviously higher than tumor samples (Figure 1A,C, Table S2). Next, we analyzed the expression levels of *UBE2C* and *PLK1* in different pathological stages and found that *UBE2C* was significantly overexpressed in stage III and IV in 17 types of cancers (Figure 1B, Table S3). *PLK1* was significantly increased in 13 types of cancer between different pathological stages (Figure 1D, Table S3). In addition, based on our previous study [42], the analysis of tumor tissue single-cell transcriptome data showed that the expressions of *UBE2C* and *PLK1* have a similar significant upregulation trend in cancer cell clusters (CSs) (Figure S1A,B). The above results suggested that the upregulated expression of *UBE2C* and *PLK1* in a variety of cancer tissues and cells is a common phenomenon. The survival analyses revealed that the high expression of *UBE2C* was significantly associated with a poor prognosis in patients of 10 cancer types (Figure 1E) while *PLK1* was also associated with 11 cancer types (Figure 1F). This suggests that *UBE2C* and *PLK1* may play important roles in the development of pan-cancer.

### 2.2. Knockdown of UBE2C and PLK1 Significantly Inhibited Cancer Cell Proliferation and Migration and Promoted Cell Apoptosis

**Figure 2:**
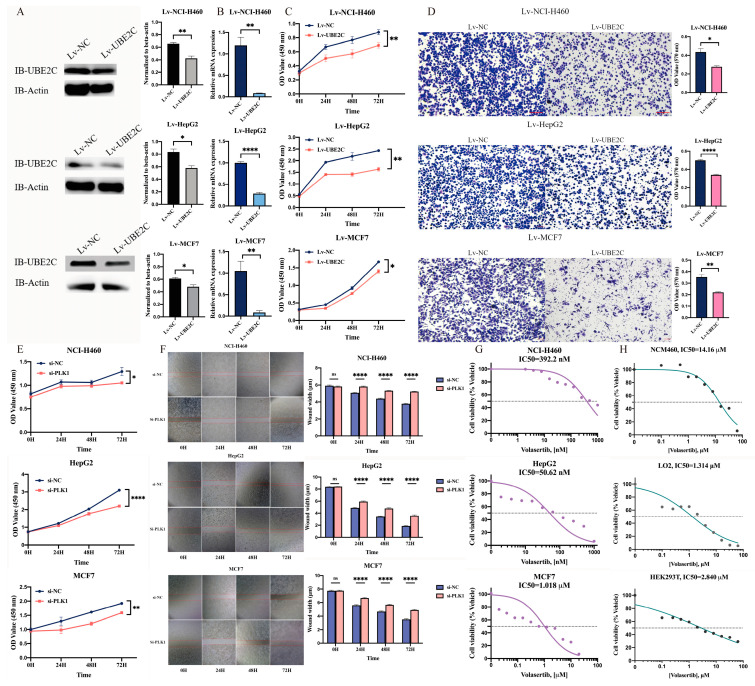
Interfering with UBE2C and PLK1 could significantly inhibit the proliferation and migration of cancer cells. (**A**–**D**) After stable knockdown of Lv-NC and Lv-UBE2C, the expression levels of UBE2C protein (**A**) and UBE2C gene copy number (**B**) as well as the cell proliferation ability (**C**) and migration ability (**D**) of NCI-H460, HepG2, and MCF7 were tested. The microscopic images were captured at 10× magnification. (**E**,**F**) After si-NC and si-PLK1 knockdown, the cell proliferation ability (**E**) and migration ability (**F**) of NCI-H460, HepG2, and MCF7 were detected. The microscopic images were captured at 4× magnification. The bar graph on the right shows that at each time point, the treatment and the control group each take at least 9 pictures of the field of view for trace width statistics. ImageJ software (version 1.53) was used to calculate the average distribution of 8 straight lines in each image, and the values indicate mean ± SEM. (**G**) Cell viability assays were measured by CCK8 assay evaluating the response of PLK1 inhibitor. NCI-H460, HepG2, and MCF7 cancer cells (**G**) as well as the NCM460, LO2, and HEK293T normal cells (**H**) are treated with different doses of volasertib with at least three biological replicates in duplicates for 24 h. The graph presents normalization of transform X of dose vs. response. ns—No significance, *: *p* < 0.05; **: *p* < 0.01; ****: *p* < 0.0001 (two-sided unpaired Student’s *t*-test).

To investigate the function of *UBE2C* on the proliferation and migration of cancer cells, we first constructed the NCI-H460, HepG2, and MCF7 cancer cell lines with the sSknockdown of UBE2C (namely Lv-NCI-H460, Lv-HepG2, and Lv-MCF7) whose expression level of the UBE2C protein (top to bottom: *p* = 0.0040, 0.0123, 0.0252) and copy number of the UBE2C gene (top to bottom: *p* = 0.0039, <0.0001, 0.0038) were significantly reduced (Figure 2A,B). The knockdown of *UBE2C* resulted in a significant loss of cell proliferation (top to bottom: *p* = 0.0095, 0.0080, 0.0381) and migration (top to bottom: *p* = 0.0139, <0.0001, 0.0037) in stable cell lines (Figure 2C,D). To explore the possible role of *PLK1* in proliferation and migration, CCK-8 and wound-healing assays after transfection with siRNA against *PLK1* for 48 h in NCI-H460, HepG2, and MCF7 cell lines were performed, respectively. The results showed that the proliferation (top to bottom: *p* = 0.0346, <0.0001, 0.0047) and migration ability (*p* < 0.0001) of the NCI-H460, HepG2, and MCF7 cells were extremely suppressed with the knockdown of *PLK1*, compared with the control group (Figure 2E,F).

Furthermore, we treated the cells with a highly selective PLK1 inhibitor, volasertib, which potently reduced cell viability in six cancer cell lines with a significantly lower IC50 value than that in normal cell lines (Figure 2G,H). These results suggest that not only the downregulation of *UBE2C* and *PLK1* but also the pharmacological inhibition of PLK1 enzyme activity can significantly inhibit the proliferation of tumor cells.

### 2.3. Combined Inhibition of UBE2C and PLK1 Mediates Greater Suppression of the Malignant Phenotype in Pan-Cancer

**Figure 3:**
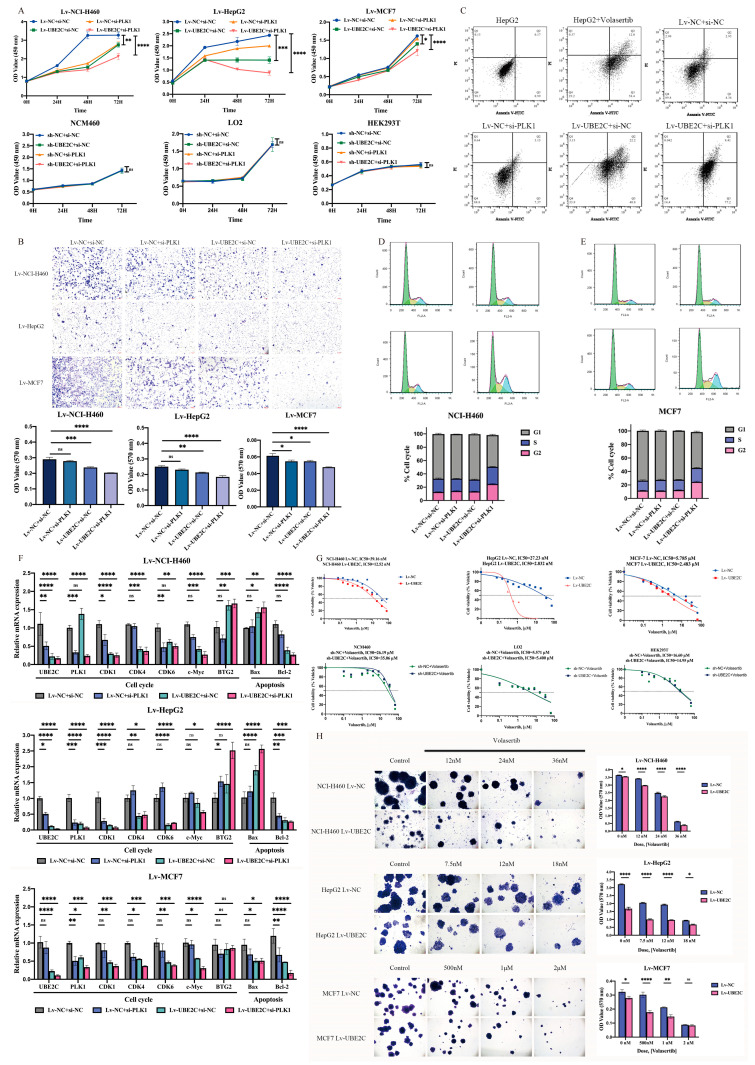
Combined inhibition of UBE2C and PLK1 could more significantly reduce the malignant phenotype of pan-cancer cells. (**A**) Co-interference with UBE2C and PLK1 significantly inhibited cancer cell (**top panel**) proliferation but not that of normal cells (**bottom panel**) at different time points. (**B**) Co-interference with UBE2C and PLK1 significantly inhibited cancer cell migration. The microscopic images were captured at 10× magnification. The bar graph on the right represents the detection of the OD value at 570 nm of crystal violet staining of the treatment group and the control group. The value represents mean ± SEM. (**C**) Co-interference with UBE2C and PLK1 significantly promoted cancer cell apoptosis. The number of apoptosis cells detected by flow cytometry. Graphical representation of % cell death (Q2+Q3) from three different experiments on HepG2 cells. (**D**,**E**) Co-interference effects of UBE2C and PLK1 on cell cycle of (**D**) NCI-H460 and (**E**) MCF7. FACS analyses were performed using si-NC or si-PLK1-transfected Lv-NCI-H460 or Lv-MCF7 cells for 24 h. Cell number (%) in each cell cycle phase is indicated in the graph. Grey color in bar graph indicates G1 phase, and blue and pink colors indicate S and G2 phases, respectively. (**F**) The expression levels of cell-cycle- and apoptosis-related genes were detected via qRT-PCR after co-interference with UBE2C and PLK1 in Lv-NCI-H460, Lv-HepG2, and Lv-MCF7 cells (from top to bottom). (**G**) PLK1 inhibitor volasertib inhibited the viability of UBE2C stable knockdown cells (**top panel**), but normal cells were not sensitive (**bottom panel**). (**H**) Combined treatment of interfering UBE2C with volasertib remarkably repressed cancer cells’ colony-forming capacity. The microscopic images were captured at 4× magnification. The bar graph on the right represents the detection of the OD value at 570 nm of crystal violet staining of the treatment group and the control group. All the values represent mean ± SEM. ns—No significance, *: *p* < 0.05; **: *p* < 0.01; ***: *p* < 0.001; ****: *p* < 0.0001 (a two-way ANOVA was used, except for (**B**), in which an ordinary one-way ANOVA was used).

To further analyze the relationship between *UBE2C* and *PLK1*, we performed a correlation analysis in tumor tissue samples across 33 cancer types, which showed that the correlation coefficient between *UBE2C* and *PLK1* was 0.30–0.93 (Figure S2, Table S4). This suggests that *UBE2C* and *PLK1* may synergistically promote tumorigenesis. To validate the effect of *UBE2C* and *PLK1* on the progress of cancer development, we transfected si-*PLK* into stable knockdown UBE2C cell lines for 72h. The results of the CCK-8 assay showed that the proliferation of co-interfering cancer cell lines was markedly suppressed compared with the control group (*p* < 0.0001) as well as the single interference group (top panel, left to right: *p* = 0.0071, 0.0002, 0.0491) (Figure 3A). The results of the transwell experiment showed that the downregulation of both *UBE2C* and *PLK1* remarkably suppressed the migration ability in pan-cancer cell lines, compared to that of single-knockdown *UBE2C* (left to right: *p* = 0.0120, 0.0274, 0.0338) or *PLK1* (left to right: *p* < 0.0001, =0.0014, 0.0338) (Figure 3B). Moreover, apoptosis detection experiments were performed after the same treatment. As a result, the number of apoptotic cells in co-interfering groups at 48 h (the total number of cells in Q2 and Q3 in the figure) was significantly higher than that of the other three groups (*p* < 0.0001) (Figure 3C). What is more, we found that the combined knockdown of *UBE2C* and *PLK1* could induce cell cycle arrest in G2 (Figure 3D,E). Correspondingly, the expression levels of the gene-related cell cycle and apoptosis detected via qRT-PCR after co-interference were significantly suppressed, such as cyclin-dependent kinase 1 (*CDK1*) (top to bottom: *p* < 0.0001, <0.0001, =0.0004), cyclin-dependent kinase 4 (*CDK4*) (top to bottom: *p* < 0.0001, =0.0135, 0.0004), cyclin-dependent kinase 6 (*CDK6*) (top to bottom: *p* = 0.0080, <0.0001, 0.0005), BCL2-associated X apoptosis regulator (*Bax*) (top to bottom: *p* = 0.0027, <0.0001, 0.0230), and BCL2 apoptosis regulator (*Bcl2*) (top to bottom: *p* < 0.0001, =0.0002, <0.0001) (Figure 3F).

In addition, we performed a CCK-8 assay using the volasertib treatment on the stable knockdown UBE2C cell lines, and there was a more sensitive response in the stable knockdown cell lines, both in terms of proliferation and their colony-forming capacity (most significant inhibition from top to bottom: *p* < 0.0001) compared with that of the control group (Figure 3G,H). This suggests that the combined inhibition of *UBE2C* and *PLK1* may more significantly suppress tumor progression by inducing cell cycle arrest and apoptosis than either of them alone.

### 2.4. UBE2C and PLK1 Promots the Development of Pan-Cancer by Influencing Cell Cycle and Metabolic Pathway

**Figure 4:**
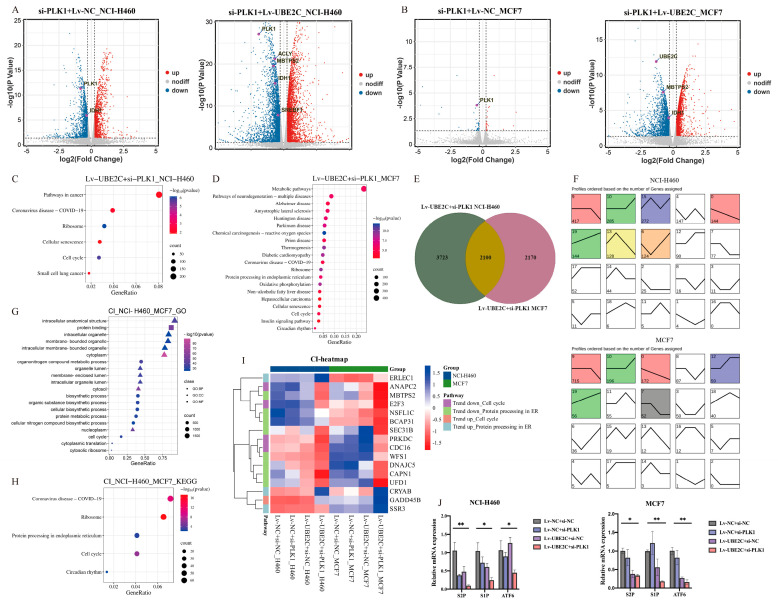
Functional enrichment analysis of RNA-seq DEGs. (**A**,**B**) The volcano maps of NCI-H460 (**left panel**: Lv-NC+si-PLK1, right panel: Lv-UBE2C+si-PLK1) and MCF-7 (**left panel**: Lv-NC+si-PLK1, **right panel**: Lv-UBE2C+si-PLK1) DEGs with threshold of |FC| > 1.2 and FDR < 0.05. (**C**) and (**D**): The KEGG pathways of NCI-H460 and MCF7 Lv-UBE2C+si-PLK1 DEGs, respectively. (**E**) Lv-UBE2C+si-PLK1 overlapping DEGs of NCI-H460 and MCF7. (**F**) The trend analyzed by gene number of (**J**) overlapping DEGs (**top panel**: NCI-H460, **bottom panel**: MCF7). The upper left corner of each rectangle: profile number. The lower left quarter of each rectangle: gene number, representing the significance of enrichment. Trend line: the gene-fitting curve in each module, and each group is an inflection point. The order from left to right is Lv-NC+si-NC, Lv-NC+si-PLK1, Lv-UBE2C+si-NC, and Lv-UBE2C+si-PLK1, respectively. Profile color: the color profile is *p* < 0.05, indicating significant enrichment. The trend is similar, and the profiles have the same color. The white profiles represent the non-significance of this expression trend. (**G**,**H**): The GO and KEGG pathways of the overall upward- and downward-trending genes, respectively. (**I**) The heatmap of cell-cycle-related and protein processing in ER-related genes from both upward and downward trends. The color represents the TPM of different genes from RNA-seq groups of NCI-H460 and MCF-7 cancer cell lines. (**J**) Expression of S1P, S2P, and ATF6 transcripts in stable knockdown cell lines of NCI-H460 and MCF7. *: *p* < 0.05; **: *p* < 0.01 (Two-way ANOVA).

To better understand the molecular signatures after the combined inhibition of *UBE2C* and *PLK1* in Lv-NCI-H460 and Lv-MCF7 tumor cells, we performed an RNA-seq analysis and analyzed the DEGs. As a result, the total DEGs in the Lv-NCI-H460 and Lv-MCF7 cancer cells were screened with |FC| > 1.2, |logCPM| > 1, and FDR < 0.05, respectively (Figure 4A,B). A study based on our previously published research suggested that *UBE2C* may promote the development of pan-cancer by influencing cell cycle through the regulation of the G1/S-phase transition [3]. A total of 2895 and 73 DEGs in Lv-NCI-H460 and Lv-MCF7 interfering with *PLK1* alone were screened, and the significant enriched pathways were cell-cycle-related, including biological regulation and DNA replication (Figure S3A–C). The results suggest that both *UBE2C* and *PLK1* may promote the occurrence and development of pan-cancer by separately influencing the cell cycle.

Moreover, to investigate thoroughly the combined inhibition effect of *UBE2C* and *PLK1*, a total of 5823 RNA-seq DEGs in the Lv-NCI-H460 and Lv-UBE2C+si-PLK1 groups were screened using the same strategy mentioned above (Figure 4A,B). In addition to the cell cycle, the DEGs in the combined inhibition were enriched in the metabolic pathways, including oxidative phosphorylation, biosynthesis, and cellular macromolecular metabolism (Figure 4C,D and Figure S3D,E). The results suggest that *UBE2C* and *PLK1* may synergistically promote tumorigenesis by affecting not only cell cycle progression but also cell metabolism.

Notably, there were 2100 overlapping genes of the Lv-UBE2C+si-PLK1 groups in the two cell lines, and a trend analysis was conducted based on the TPM of these genes in all the RNA-seq groups (Figure 4E). The results showed that, compared with the control and the single interference groups, the DEGs with an increasing expression trend were enriched at profiles 10 and 19 and the DEGs with a decreasing expression trend at profile 0 and 9 (Figure 4F). As expected, the significant enriched pathways were the cell cycle and metabolic process, including protein processing in the endoplasmic reticulum (ER) (Figure 4G,H). Specifically, we noticed the genes that involved the cell cycle and protein processing in the endoplasmic reticulum, the expressions of cell division cycle 16 (*CDC16*), E2F transcription factor 3 (*E2F3*), and membrane-bound transcription factor peptidase site 2 (*MBTPS2*, as known as *S2P*) were significantly reduced with the combined inhibition with *UBE2C* and *PLK1*, compared with either the control or knockdown *UBE2C* or *PLK1* alone (Figure 4I). The S2P protease functions in the signal protein activation that is involved in the sterol control of transcription and the endoplasmic reticulum stress response. The expression levels of genes related to the ER stress response, including *S2P*, membrane-bound transcription factor peptidase site 1 (*S1P*), and activating transcription factor 6 (*ATF6*), were detected via qRT-PCR in the Lv-MCF7 and Lv-NCI-H460 cells. The results showed that the expression of *S1P* (left to right: *p* = 0.0204, 0.0199), *S2P* (left to right: *p* = 0.0048, 0.0031), and *ATF6* (left to right: *p* = 0.0373, 0.0023) in the co-interference group was significantly decreased compared to that in the negative control group (Figure 4J).

To identify accurately how cell-cycle-related genes affect tumorigenesis by influencing metabolic processes, we also performed an RNA-seq analysis after the combined inhibition of *UBE2C* and *BIRC5* simultaneously. In fact, in our previous research, based on big data mining technology, we screened for cell-cycle-related candidate genes: *UBE2C* and *PLK1* as well as *BIRC5*. We performed a correlation analysis in tumor tissue samples across 33 cancer types, which showed that the correlation coefficient between *UBE2C* and *BIRC5* was 0.23–0.94 (Figure S4). It also seems to suggest that *UBE2C* and *BIRC5* may synergistically promote tumor progression. We also performed a similar phenotype study on BIRC5 as described in our unpublished research data, and the results of the RNA-seq analysis after the knockdown of BIRC5 alone or the knockdown of both UBE2C and BIRC5 in Lv-NCI-H460 and Lv-MCF7 cells showed that the DEGs were significantly enriched in the cell cycle and metabolic pathways (Figure S3F–M). Our data suggest that there is an added benefit of the combined inhibition of *UBE2C* and *PLK1* vs. either *BIRC5* alone or *UBE2C* and *BIRC5*’s combination. However, based on the RNA-seq results, we were surprised to find that the combined inhibition of *UBE2C* and *BIRC5* may also affect the cell cycle and metabolic progression as well as the combination of *UBE2C* and *PLK1*. The results indicated that this may provide a theoretical foundation for the analysis and screening of candidate targets.

### 2.5. Combination of RNA-Seq Analysis and Bioinformation for Target Discovery Reveals Promising Candidates IDH1/ACLY

**Figure 5:**
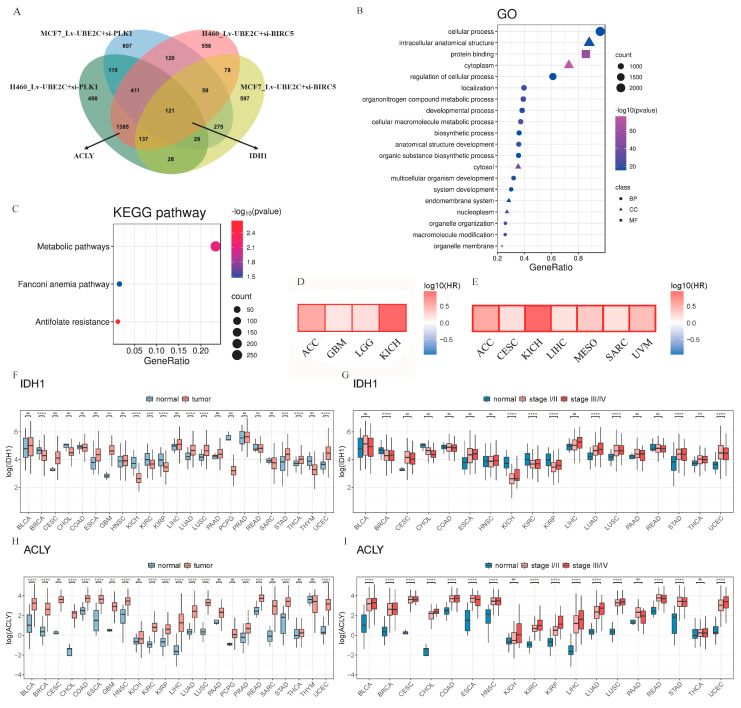
Promising candidates, IDH1/ACLY, were discovered from the combination of RNA-seq analyses. (**A**) The overlap DEGs of four combined interference groups. (**B**,**C**), The GO and KEGG pathways of (**A**) two and more overlapping DEGs, respectively. (**D**) Kaplane–Meier estimates of overall survival (OS) are shown according to the expression level of IDH1. (**E**) Kaplane–Meier estimates of overall survival (OS) are shown according to the expression level of ACLY. (**F**) The expression levels of IDH1 in normal tissues and tumor samples. (**G**) The expression levels of IDH1 in pathological stages. (**H**) The expression levels of ACLY in normal tissues and tumor samples. (**I**) The expression levels of ACLY in pathological stages. ns—no significance; **: *p* < 0.01; ***: *p* < 0.001; ****: *p* < 0.0001, (two-sided unpaired Student’s *t*-test).

As demonstrated above, we screened promising candidates in four sets of RNA-seq data, with the co-interference of *UBE2C* and *PLK1* and the co-interference of *UBE2C* and *BIRC5* in Lv-NCI-H460 and Lv-MCF7, respectively. The DEGs were screened with logFC < −1.2, FDR < 0.05, and |logCPM| >1, respectively. There were 2755 overlapping genes in two or more groups (Figure 5A). As expected, the DEGs were significantly enriched in metabolic-related pathways (Go: cellular macromolecule metabolic process, *p* = 4.03 × 10^−28^, KEGG: metabolic pathways, *p* = 0.0070) (Figure 5B,C). The results suggest that the synergistic effect of the cell-cycle-related genes *UBE2C*, *PLK1,* and *BIRC5* may promote the development of pan-cancer by affecting cell metabolism.

Next, we focused on the genes that were enriched in metabolic pathways, including *IDH1*, which appeared in the overlapping genes of four groups. Bioinformatics analyses were performed using the TCGA database. The result showed that the expression of *IDH1* was higher in 10 types of tumors than in normal tissues, especially in the brain, lung, stomach, and endometrium (Figure 5F). We analyzed the expression levels of *IDH1* in pathological stages and found that it was significantly overexpressed in stage III and IV in nine types of cancers (Figure 5G). Moreover, according to our previous study [42], the analysis of our tumor tissue single-cell transcriptome data showed that the expression of IDH1 has a significant upregulation trend in cancer cell clusters (CSs) (Figure S1C). The survival analyses revealed that the high expression of *IDH1* was significantly associated with a poor prognosis in patients with four cancers (Figure 5D).

Additionally, we noticed another gene, *ACLY*, that appeared in the overlapping genes of Lv-NCI-H460, Lv-UBE2C+si-PLK1, and Lv-UBE2C+si-BIRC5. The result of our bioinformatics analyses showed that it appeared to be more promising as a therapeutic target, because its expression was higher in 16 types of tumors than in normal tissues while it was significantly overexpressed in stage III and IV in 15 types of cancers (Figure 5H,I). The expression of *ACLY* has a significant upregulation trend in CS5 of CRC, CS2 of LC, CS3 of OV, CS3 of PDAC, and CS4 of SCC in the tumor tissue single-cell transcriptome (Figure S1D). The survival analyses revealed that the high expression of the *ACLY* was significantly associated with a poor prognosis in patients with seven cancers (Figure 5E). This suggests that *ACLY* may not be underestimated in the development of pan-cancer.

Apart from this, we performed a qRT-PCR test to detect the expression of the *IDH1* and *ACLY* genes in Lv-NCI-H460, Lv-HepG2, and Lv-MCF7 cells. Compared with the control and single interference, the expressions of both *IDH1* and *ACLY* were significantly decreased. The above results suggested that the upregulated expression of *IDH1* and *ACLY* in a variety of cancer tissues and cells is a common phenomenon and both may be potentially regulated by UBE2C, PLK1, and BIRC5.

### 2.6. Evaluation of the Effect of Using Inhibitors Alone or in Combination on Pan-Cancer

Since there are no highly selected inhibitors for UBE2C currently, we decided to perform an in vitro cell viability assay with the PLK1 inhibitor volasertib (Figure 3G), the IDH1 inhibitor ivosidenib, the ACLY inhibitor bempedoic acid, and the BIRC5 inhibitor YM155 in treating stable knockdown *UBE2C* cell lines for 24 h. The treatment with ivosidenib induced a 50% growth inhibition in Lv-HepG2 and Lv-UBE2C with a much lower IC50 than that of Lv-NC (Figure S5A). Additionally, the ACLY inhibitor bempedoic acid showed potent anti-tumor activity against all stable cell lines 24 h after the combined treatment, and a significantly lower rate of decrease in IC50 was observed in Lv-MCF7 (Figure S5B). Furthermore, YM155 showed strong antitumor activity in Lv-H460 cells at an extremely low concentration combined with UBE2C knockdown. In addition, normal epithelial cell lines were treated with ivosidenib and bempedoic acid, respectively, and the cell viability measurements showed that the normal epithelial cell lines achieved a 50% killing activity at higher concentrations than those of cancer cell lines (Figure S5C).

Next, we investigated the in vitro efficacy of combining volasertib with YM155. The combination treatment of volasertib+YM155 exhibited synergistic anti-tumor activity in the in vitro cell viability assay, although this synergy was achieved at a much lower IC50 than that of volasertib (Figure 2G and Figure S5D), though higher than that of YM155. In addition, YM155 had a killing activity of about 50% against normal cells at a concentration lower than that of the combined treatment whereas volasertib did not (Figure 2H and Figure S5E). Based on toxicity and safety concerns, this combination may seem less effective than expected.

**Figure 6:**
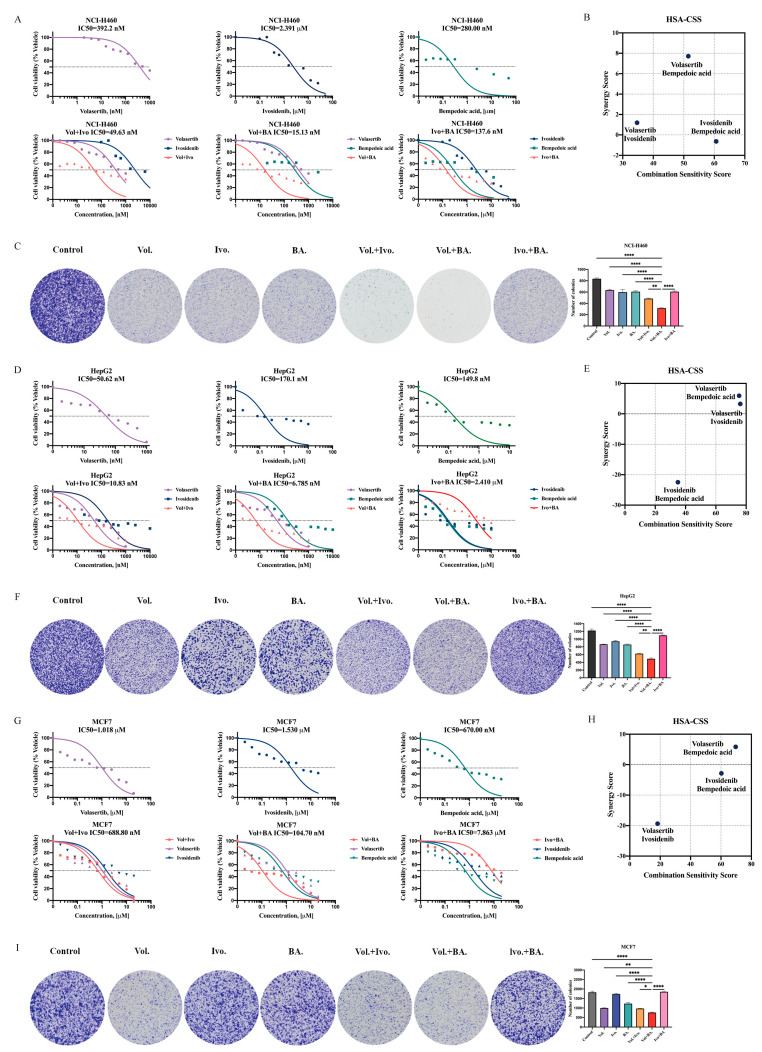
PLK1 and ACLY inhibition imparts synergistic anti-proliferative response against pan-cancer cells. (**A**,**D**,**G**) Cell viability assays evaluating the response of PLK1, IDH1, and ACLY inhibitors. Percentages of live NCI-H460, HepG2, and MCF7 cells treated by different doses of PLK1 inhibitor volasertib and/or IDH1 inhibitor ivosidenib and/or ACLY inhibitor bempedoic acid are shown as the mean of at least three biological replicates in duplicates. The graph presented as the normalization of transform X of dose vs. response. (**B**,**E**,**H**) The highest single agent (HSA) model assesses the extent of drug–drug cell line synergies. The synergy scores or combination sensitivity scores (CSSs) shown in this plot are the summarized scores for whole combination matrix. (**C**,**F**,**I**) Representative images of colony formation assays. NCI-H460, HepG2, and MCF7 cells treated with volasertib and/or ivosidenib and/or bempedoic acid by corresponding 1/2 Vol+BA IC50 dose concentration followed by colony staining using crystal violet of at least three biological replicates in duplicate. The bar graph on the right represents the mean ± SEM in each group of samples. * *p* < 0.05, ** *p* < 0.01 and **** *p* < 0.0001 (ordinary one-way ANOVA). Vol: Volasertib; Ivo: Ivosidenib; BA: Bempedoic acid.

Next, the cytotoxicity of ivosidenib and bempedoic acid in combination and their combination with volasertib, respectively, was assessed in a set of NSCLC cell lines (NCI-H460, A549, and H520). As previously described, the cells were treated with the indicated inhibitors for 24 h to assess the monotherapy-induced and combination effect. The combination of ivosidenib and bempedoic acid showed nearly no relevant synergistic inhibition of cell viability compared to ivosidenib or bempedoic acid separately (Figure 6A and Figure S6A,B). In contrast, the combination of volasertib and ivosidenib or volasertib and bempedoic acid significantly increased the cytotoxic effect in three NSCLC cell lines (except for A549) (Figure 6A and Figure S6A,B). Remarkably, the combination of volasertib+bempedoic acid showed stronger cytotoxicity than volasertib+ivosidenib, which is reflected by the high synergy score of the volasertib+bempedoic acid combinations (Figure 6B and Figure S6C,D). As expected, the combination of volasertib+bempedoic acid exhibited the strongest inhibitory effect on the colony formation ability of the NSCLC cell lines, compared with the control (*p* < 0.0001) and the mono or combination therapy (NCI-H460 Vol.+BA. vs. Vol.+Ivo., *p* = 0.0011; A549 Vol.+BA. vs. Vol.+Ivo., *p* = 0.0001) (Figure 6C and Figure S6E).

To further validate the combined effect in HCC cell lines, the same treatment as described above was used for verification in HepG2, Sk-hep-1, and SNU-387 cells. In HepG2 and Sk-hep-1, the combination effect of volasertib and bempedoic acid was more dominant and had higher synergy scores compared to those of monotherapy and other combinations (Figure 6D,E and Figure S7A,B). However, although all three combinations of SNU-387 showed synergistic effects, the combination of ivosidenib and bempedoic acid was more effective than the other two HCC cell lines (Figure S7C,D). Moreover, the treatment of volasertib and bempedoic acid more significantly inhibited the colony-forming ability of HepG2 (Vol.+BA. vs. Vol.+Ivo., *p* = 0.0060) and Sk-hep-1 cells (Vol.+BA. vs. Vol., *p* = 0.0024), compared to ivosidenib and bempedoic acid inhibiting the ability of SNU-387 cells (Ivo.+BA. vs. Vol.+BA., *p* < 0.0001) (Figure 6F and Figure S7E,F).

The combination effect that follows is illustrated in breast cancer cell lines. As a result, in MCF7 and HCC1937, only the combination of volasertib and bempedoic acid showed a better synergistic inhibition of cell viability and higher synergy scores (Figure 6G,H and Figure S6F,G), as well as a stronger inhibition of colony-forming ability (MCF7 Vol.+BA. vs. Vol.+Ivo., *p* = 0.0212; HCC1937 Vol.+BA. vs. Vol.+Ivo., *p* = 0.0160) (Figure 6I and Figure S6H). In addition, all combinations were well-tolerated in normal epithelial cell lines, with much higher IC50 scores than those in cancer cell lines (Figure S8). Above all, their combination caused a significant reduction in cell growth compared to the drugs alone; the potential therapeutic effects of volasertib and bempedoic acid’s combination may be more promising in treating pan-cancer.

**Figure 7:**
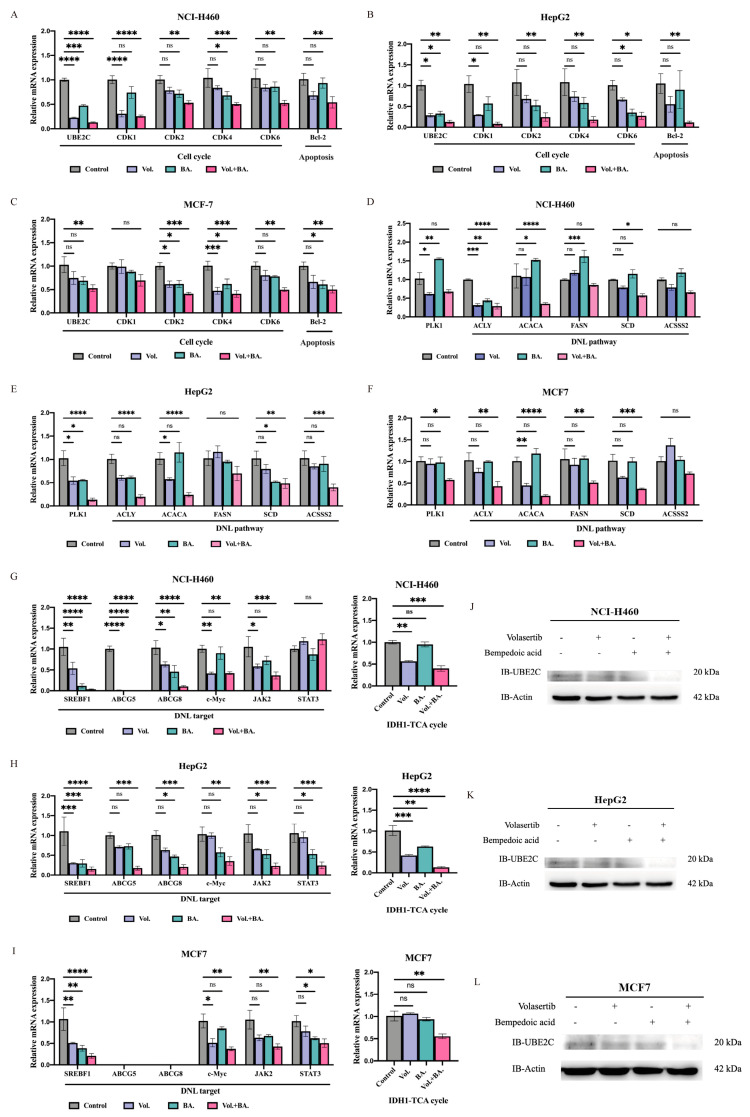
Volasertib combined with bempedoic acid suppressed expressions of cell−cycle−related and DNL−related genes. (**A**,**D**,**G**) NCI-H460 cells, (**B**,**E**,**H**) HepG2 cells, and (**C**,**F**,**I**) MCF7 cells were treated with volasertib and/or ACLY inhibitor bempedoic acid by corresponding 1/2 IC50 dose concentration for 24 h. Expressions of genes were evaluated via real-time PCR. (**J**) NCI-H460, (**K**) HepG2, and (**L**) MCF7 cells were treated with volasertib and/or bempedoic acid for 24 h, and relative protein expression of UBE2C was determined by Western blotting. ns—no significance, * *p* < 0.05, ** *p* < 0.01, *** *p* < 0.001, and **** *p* < 0.0001 (two-way ANOVA) indicate significant differences between fold induction.

Based on these results, we hypothesize that their combination may more strongly affect the cell cycle to treat pan-cancer. After treatment with the indicated dose of volasertib and bempedoic acid alone or in combination, qRT-PCR experiments were performed on related mRNA to study the mechanism inhibiting cell proliferation. The results showed that the combined treatment inhibited the expression of cyclin-dependent kinase 2 (*CDK2*) at the mRNA level (*p* < 0.05) in both pan-cancer cell lines (Figure 7A–C and Figure S9A–D). Since ACLY is the key regulator between the high rates of aerobic glycolysis and DNL, which is exhibited in many types of tumor cells, it is possible that bempedoic acid may influence the changes in DNL pathway molecules. To explore whether the strongly proliferative inhibition induced by the combined application is associated with DNL pathway-related genes in pan-cancer cell lines, we further investigated the mRNA levels of related genes. In the combination group, the mRNA levels of acetyl-CoA carboxylase alpha (*ACACA*) and sterol regulatory element binding transcription factor 1 (*SREBF1*) were more significantly decreased than those in the monotherapy and control groups (*p* < 0.0001) (Figure 7D–F and Figure S9E–H). In most of the cell lines tested, we were surprised to find that the mRNA expression levels of cell-cycle-related *UBE2C* and *CDK4*, DNL downstream-related Janus kinase 2 (*JAK2*), and *IDH1* were also significantly decreased (*p* < 0.01) (Figure 7A–C,G–I and Figure S9A–D,I,L). Furthermore, the protein expression of UBE2C also decreased with the combination treatment (Figure 7J–L).

## 3. Discussion

Cell cycle dysregulation is a distinctive feature of many cancers; therefore, cell cycle regulators are considered attractive targets in cancer therapy [43]. UBE2C is required to facilitate the APC/C-mediated ubiquitylation of cyclin B1 and securin [6,44]. The ubiquitylation of cell cycle proteins in this manner leads to their degradation and is a major control point in the metaphase to anaphase transition as well as the mitotic exit [45]. However, it seems there is little evidence of this ubiquitination degradation involving PLK1, although PLK1 plays a key role in the cell cycle progression of mitosis through its effects on chromosome separation, spindle assembly, and cytokinesis [46]. In this study, we found that *UBE2C* and *PLK1* were significantly highly expressed in more than 13 cancer tissues. As important components of the cell cycle, high levels of *UBE2C* and *PLK1* are commonly associated with aggressive cancers and poor patient prognoses for multiple cancer types. Additionally, we found that the knockdown of *UBE2C* and *PLK1*, respectively, can reduce the proliferation and migration capacity, and induce the cell cycle arrest and apoptosis of pan-cancer cell lines, which was consistent with the results of previous studies [7,16,47]. Our data suggest that combined inhibition of *UBE2C* and *PLK1* can more significantly strongly attenuate the proliferation, migration, colony formation and other malignant phenotypes of pan-cancers while inducing more intense cell cycle arrest and apoptosis. This molecular mechanism may be attributed to the significant decrease in *CDK1* and the *CDK4/6* mRNA expression observed after the co-knockdown of *UBE2C* and *PLK1*. CDK1 is a cyclin-dependent kinase (CDK) essential for cell cycle progression [48]. During the G2 phase, CDK1 binds to and becomes activated by cyclin A2 and cyclin B. After entering the mitotic phase, B-type cyclins are degraded in late mitosis by APC/C [49], which inhibits CDK1 activity and enables chromosome segregation as well as mitosis and cytokinesis [50]. For UBE2C, it has been shown that its protein is involved in the ubiquitin-mediated degradation of cyclin A and B [51], and the loss of *UBE2C* may result in the inability of APC/C to assemble and, in turn, to fail to trigger the ubiquitination degradation of cyclins, thereby affecting mitotic progression. Moreover, PLK1 has an important role in the activation of cyclin B-CDK1 complexes by at least two mechanisms [52,53]. In addition, when stimulated by mitogenic signals, the cyclin D-CDK4/6 complexes phosphorylates various targets, particularly the retinoblastoma tumor suppressor protein (RB, encoded by *RB1*). Upon the phosphorylation of RB, E2F transcription factors can activate the transcription of numerous genes that promote the S-phase, including those involved in the further phosphorylation of RB. This initiates a positive feedback loop that facilitates cell cycle progression. The CDK4/6-RB pathway is abnormally activated in a variety of cancers, characterized by the abnormal amplification of genes related to the pathway and resistance to inhibition by cyclin-dependent kinase inhibitors (CKIs), including members of the INK4 family (p16^INK4A^, p15^INK4B^, p18^INK4C^, and p19^INK4D^) [43]. A potential inverse correlation between the expression or activation of pro-proliferative PLK1 and pro-apoptotic p16 has been demonstrated in several cancer studies [54,55]. In some mouse tumor models with specific genetic backgrounds, the loss of *CDK4* and *CDK6* impedes tumor progression [56,57]. Given that the regulatory role of UBE2C and PLK1 in the cell cycle may be due to the downregulation of *CDK1* and *CDK4/6*, along with their aberrant activation and positive correlation in pan-cancer tissues, coupled with their involvement in the manifestation of pan-cancer phenotypes, targeting both UBE2C and PLK1 may represent a promising therapeutic strategy. However, there remains a significant lack of understanding regarding how their downstream molecular cascades or networks are rewired in human malignancies.

To accurately elucidate the possible regulatory mechanism of their combined inhibition, an RNA-seq analysis of the combination of knockdown *UBE2C* with *PLK1* or *BIRC5* was performed. By analyzing and screening the transcriptome data, we found that the combined inhibition of cell proliferation may affect metabolic pathways by decreasing the expression of *IDH1* and *ACLY*. Moreover, by evaluating the effect of their use alone and combined with several inhibitors, we found that volasertib combined with bempedoic acid showed the strongest synergistic anti-proliferative activity in pan-cancer cell lines, which may decrease the mRNA expression of *ACACA* and *SREBF1* by disrupting de novo lipid synthesis. Tumor cells rely on de novo fatty acid synthesis for growth and proliferation and as such, are expected to be vulnerable to the inhibition of fatty acid synthetic enzymes. Previous studies have provided comprehensive evidence that oncogene *MYC*, in collaboration with the transcription factor SREBP1 (coded by *SREBF1*), regulates fatty acid synthesis to promote tumorigenesis [58]. ACLY is necessary for tumorigenesis in mouse models of cancer, and the tool compound inhibitors of ACLY with high IC50 values have been reported to have anti-tumor efficacy in xenograft models of lung and prostate cancer [59]. In this study, after the pharmacological combined inhibition of PLK1 and ACLY, the mRNA expression levels of *SREBF1* and *MYC* were significantly decreased in most pan-cancer cell lines. *ACLY* transcription is promoted by SREBP1 [60], but the significant decrease in *ACLY* mRNA expression in some of the cancer cells observed in the combined treatment may not completely be due to SREBP1. It may be due to the fact that the mRNA of *UBE2C* and *PLK1* in the combined treatment declined significantly, which is consistent with our transcriptome data. Although there is no direct evidence that UBE2C may be a target for the phosphorylation of PLK1, PLK1 has a conserved role in liberating the APC/C from its inhibitors [61]. The precise control of PLK1 activity is crucial to maintaining APC/C activation under the control of the spindle assembly checkpoint [62]. Therefore, the mechanism of decreased *UBE2C* expression after the combined inhibition of PLK1 and ACLY remains to be further explored. In addition, a significant reduction in *CDK2* mRNA expression was observed across all tested pan-cancer cell lines upon the pharmacological co-inhibition of PLK1 and ACLY. This finding may offer novel insights into the cell cycle arrest mechanism that cannot be solely attributed to *CDK1* inhibition as previously reported. The activation of CDK2 occurs through its association with E- or A-type cyclins, and it is essential for cells to progress into the S-phase [43]. Although the requirement of CDK2 activity in tumor initiation and maintenance is still not clear [43], several studies have demonstrated that the depletion of *CDK2* inhibits cancer cell growth and disrupts cell cycle progression [63,64,65]. To support this speculation about co-regulation by PLK1 and ACLY, more experimental evidence is needed in the future.

Targeted drug combination therapy is a prospective clinical therapeutic strategy that could induce apoptosis and cell cycle arrest by regulating some targeted proteins and signaling pathways. Previous studies have revealed that the interference of *UBE2C* inhibits the growth of cancer cells and blocks the G2/M transition, triggering apoptosis [47,66,67]. As an important kinase of progression through the S-phase and from the G2-phase into mitosis (M-phase), multiple studies have revealed that the inhibition of PLK1 induced a mitotic catastrophe, followed by immediate cell death via apoptosis and necroptosis [68,69]. The oncogene, anti-apoptotic factor *BIRC5*, which was found to be targeted by different inhibitors, induced tumor cell apoptosis and cell cycle arrest [70,71,72]. Our study found that the combined knockdown of *UBE2C* with *PLK1* significantly inhibited tumor cellular proliferation and triggered more lethal apoptosis and cell cycle arrest, compared to those of the knockdown of either alone. Our results suggest that it may be induced by the significantly decreased expression of cyclin-dependent kinases and *BCL-2* after the co-interference, and apoptosis has been recognized as a critical intracellular process that maintains organism homeostasis and controls cell population [73]. Interestingly, the downregulation of *MBTPS2*, a protease that plays a role in the activation of signal proteins involved in sterol control transcription and ER stress responses, was found in four groups of co-interfering RNA-seq data. This suggests that there may be a regulatory mechanism between *UBE2C*, *PLK1*, *BIRC5,* and *MBTPS2*. *MBTPS2*, as known as S2P, was discovered as a protein essential for the proteolytic activation of the transcription factor SREBP [74]. Previous data indicate that S2P is also required for the activation of other membrane-bound transcription factors such as ATF6, an inducer of chaperone expression in the mammalian unfolded protein response (UPR) [75]. Folding factors assist newly synthesized proteins in acquiring the correct three-dimensional fold, and a complex quality control system guarantees that only correctly folded proteins exit the ER. When misfolded proteins accumulate, the UPR is induced and ATF6 is activated after subsequent cleavage by S1P and S2P [76,77]. The activated ATF6 is released from the Golgi membrane and, upon transport into the nucleus, induces the expression of ER stress gene products [76,78]. Some studies have provided evidence that the decreases in S2P expression, and the absence of mature SREBP1 and ATF6 production, resulting in the failure to activate the UPR, can induce apoptosis in liposarcoma and prostate cancer cells [79,80]. A more recent study has found that the knockdown of *MBTPS2* expression in human prostate cancer cells impaired cholesterol synthesis and uptake and reduced the expression of key regulators of fatty acid synthesis, FASN and ACACA, through SREBP signaling [77]. These data suggest that tumors driven in different key regulatory components of DNL and the UPR might respond dramatically to *MBTPS2* inhibition. Our data suggest that either the combined inhibition of different targetable proteins associated with mitosis, including UBE2C, PLK1, and BIRC5, or the combination of volasertib with bempedoic acid may significantly inhibit the mRNA expression of *MBTPS2*, resulting in metabolic disorders, inducing the apoptosis of tumor cells, and contributing to the inhibition of *SREBF1* expression. Apoptotic cells and changes in lipid synthesis in different pan-cancer cells need to be specifically observed, and more evidence is needed for whether these changes are caused by the inhibition of cell-cycle-related proteins followed by the inhibition of *MBTPS2* and *SREBF1* expression.

DNL is fundamental for the survival of multicellular organisms [81]. DNL is constitutively active in many cancer cells and contributes most of the intracellular lipid mass [82]. The critical role of DNL enzymes in regulating tumorigenesis has been shown through genetic evidence from several studies including the knockdown of *ACLY* [83,84], *ACC* [85], *FAS* [86], and *SCD* [87] in various tumor types [60,88]. Indeed, several HCC studies have focused on DNL inhibition as a therapeutic option, but such work remains in its infancy [89,90]. The data described in the present study showed that the combined inhibition *UBE2C* and *PLK1* or *BIRC5* caused a decrease in the mRNA expression of *ACLY*, suggesting a possible novel strategy for cancer therapy. The enzyme ACLY is considered to be the key regulator in the initial step of de novo lipid synthesis, playing a crucial role in controlling the rate of this process [91]. DNL inhibitors have entered clinical-stage development and may become the foundation for a new class of therapeutics. Bempedoic acid as an ATP citrate lyase inhibitor, which reduces low-density lipoprotein (LDL) cholesterol levels [92], has been demonstrated to inhibit hepatocellular carcinoma in mice [84]. A recent study took a similar approach; bempedoic acid in combination with cell cycle kinase inhibitors (palbociclib targeting CDK4/6) reduced cell viability and invasion in a panel of breast and pancreatic cancer cell lines [93]. Our present study first examined the effects of the pharmacological combined inhibition of PLK1 and ACLY on the cell proliferation and clone formation of several cancer cell types. It may be possible to obtain a stronger synergistic anti-tumor activity resulting in a decreased expression of *JAK2*, in addition to the transcriptional regulation of *ACLY* by SREBP1. The inhibition of the JAK2-STAT3 pathway, which enhances the ROS accumulation and triggers apoptosis, has been reported in several studies [94,95]. However, what role the downregulated *JAK2* pathway may play needs further research.

Bypassing DNL inhibition/compensatory pathways is another key concern for single DNL inhibitors. ACSS2 catalyzes the reaction of acetate and CoA to form acetyl-CoA, which supplies lipogenic acetyl-CoA independently of ACLY, and is subsequently used for fatty acid biosynthesis [96,97]. The inhibition of ACLY can be bypassed in some cancer cells [98] and in the livers of mice fed a high-fructose diet [96] through the upregulation of ACSS2. With hypoxia or a citrate/isocitrate carrier deficiency, another alternative pathway for DNL is the reductive carboxylation of glutamine via IDH1 and mitochondrial cytosolic isocitrate dehydrogenase 2 (IDH2) [99,100]. In this study, together with the inhibition of the classical DNL pathway, we found that the scavenging of alternative carbon sources, through the downregulation of Acetyl-CoA synthetase 2 (ACSS2) and IDH1 after combination therapies, may comprehensively target the DNL pathway in a therapeutic setting. But at the same time, we found that the other two inhibition combinations, PLK1 combined with IDH1 and IDH1 combined with ACLY, were less effective in inhibiting the malignant phenotype of pan-cancer cell lines. We suspected that the reason may be that the IDH1 inhibitor treated, ivosidenib, mainly targets the mutated IDH1 or that pan-cancer cells may take advantage of alternative pathways for lipid synthesis during this inhibition. The data generated in clinical settings with respect to ACLY inhibition lowered the established LDL safely and effectively [101,102]. However, further comprehensive investigations into the therapeutic efficacy and safety of this combined approach for the long-term inhibition of DNL and cell cycle in diverse malignancies are likely to hold significant clinical implications.

Previous studies have shown that the pharmacological inhibition of both PLK1 and BIRC5 synergistically compromises the viability of *p53*-mutated HCC cells in vitro and in vivo by the combination treatment of volasertib with YM155 [103]. However, a recent study showed that the ability of YM155 to suppress the growth of racially distinct TNBC xenografts was weaker than that of the combination of volasertib and barasertib (AURKB inhibitor), due to the different phosphorylation of BIRC5 by PLK1 and AURKB [104]. In this study, strong synergistic anti-proliferation effects of volasertib and YM155 were observed in *p53* wild-type HepG2 cell lines, but it appears YM155 may also be highly toxic to normal epithelial cell lines. This dual effect is possibly due to YM155 via the transcriptional inhibition of the *BIRC5* gene promoter; meanwhile, the phosphorylation of BIRC5 at Ser20 by PLK1 kinase is essential for accurate chromosome alignment and cell proliferation [105], and volasertib, as a typical kinase domain inhibitor, greatly inhibits PLK1 function [28]. Whether the combination of YM155 with volasertib targeting other cell types and organ systems becomes widely adopted for treating other tumor indications safely and effectively remains to be determined. Although this study was performed in vitro, the mechanistic elucidation of a promising target in pan-cancer is necessary to fully utilize pan-cancer cell models and thus explore new areas of drug applications for preclinical evaluation, as has been well summarized in previous examples [106]. Indeed, more evidence is needed to clarify the exact mechanism for relevant therapeutic effects in various organoid and animal models.

**Figure 8:**
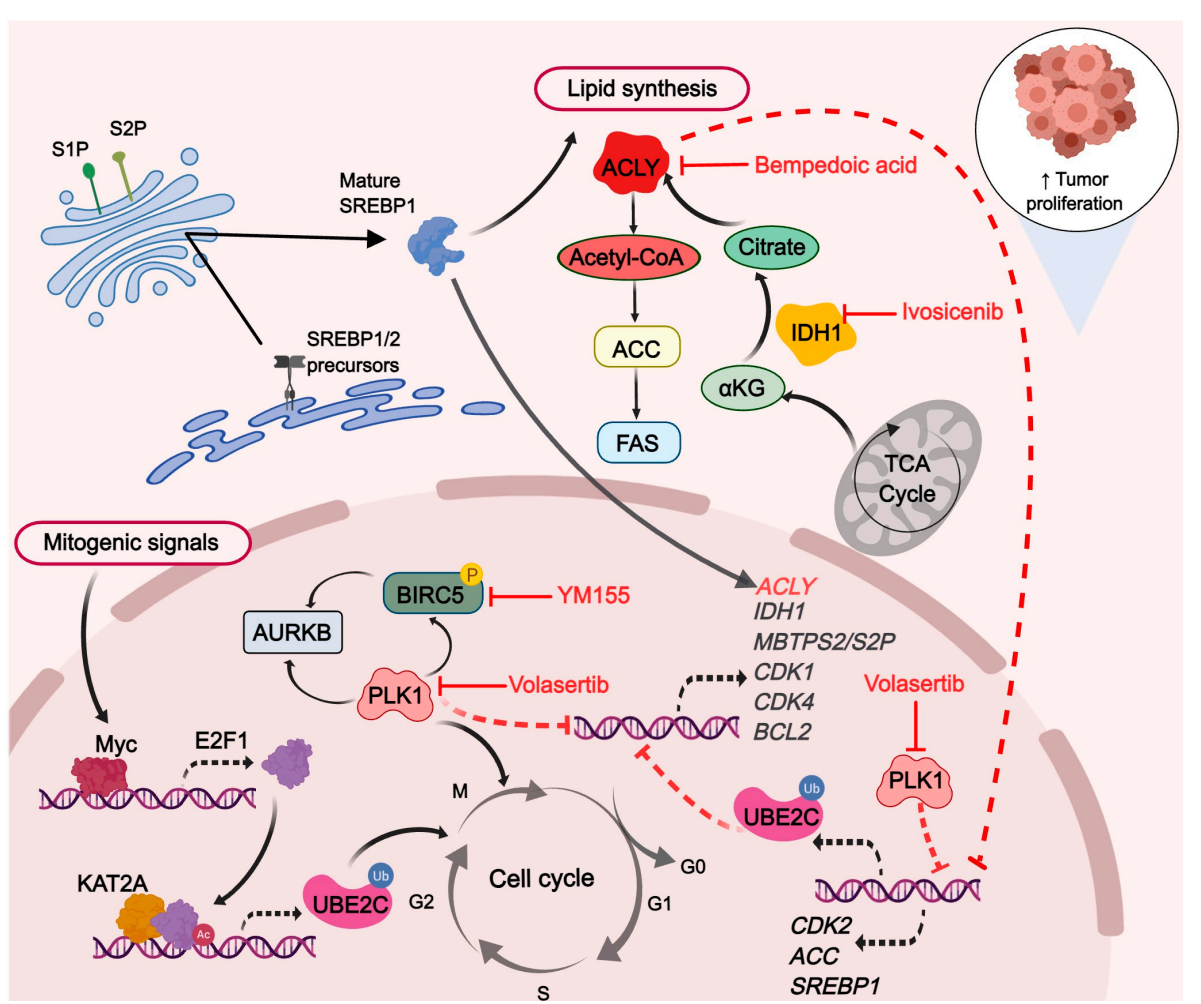
ACLY via lipid synthesis coordinates the combined UBE2C and PLK1 inhibition cell cycle arrest response. The black solid arrows depict processes in which a protein or enzyme has been implicated. The black dashed arrows represent transcription facilitation. The red solid inhibitory arrow signifies the inhibitors effect. The red dashed inhibitory arrow indicates transcriptional repression.

In summary, we identified a new UBE2C/PLK1-ACLY axis that plays an essential role in the cell cycle and pan-cancer cells (Figure 8). The deficiency of UBE2C and PLK1 largely abrogated lipogenesis and tumorigenesis by decreasing ACLY expression. The combined pharmacological inhibition of ACLY and PLK1 may inhibit pan-tumor cell proliferation through UBE2C to achieve a “triple win”. The targeting of ACLY and PLK1, or alternatively, the simultaneous targeting of other dysregulated metabolic pathways while starting from cell-cycle-related proteins, presents novel prospects for cancer treatment. Future studies will be needed to investigate whether this combination strategy would be effective and safe in PDX models and, more importantly, whether it would provide potential clinical benefits.

## 4. Materials and Methods

### 4.1. Data Source

Expression data and corresponding clinical information of different kinds of cancer patients were downloaded from the Cancer Genome Atlas (TCGA, https://portal.gdc.cancer.gov, accessed on 26 December 2022).

### 4.2. Transcriptional Expression of Different Genes in Pan-Cancer

The analysis of gene expression and the identification of differentially expressed genes were performed by comparing the expression profiles of cancerous vs. normal samples within the same patient in a paired analysis. The expression profiles of 23 tumor entities in TCGA were analyzed in EdgeR Bioconductor package version 4.0.3. Results with an FDR ≤ 0.01 were considered significant.

### 4.3. Pathological Staging Expression Analysis

The TPM expression of *UBE2C* and *PLK1* at different pathological stages in the TCGA database were represented by box plots, and the Student’s *t*-test was employed to compare the relative expression levels among different pathological stages. *p* < 0.05 indicated statistically significant differences.

### 4.4. Prognostic Analysis of Patients in Pan-Cancer

The clinical outcome of patients with different types of cancers was determined using the Kaplan–Meier survival curves. For the overall survival (OS), the samples were divided into two groups according to the median expression of the mRNAs (high vs. low). With the use of GEPIA 2 (http://gepia2.cancer-pku.cn/#index, accessed on 5 January 2023), Kaplan–Meier survival analysis and the log-rank test were employed to compare OS between the tumor and normal cohorts. *p* < 0.05 indicated statistically significant differences.

### 4.5. Spearman’s Correlation among UBE2C and PLK1

Spearman’s correlation coefficient analysis was performed to explore the correlation among UBE2C and PLK1 in 33 cancers with more than 200 tumor tissues from TCGA database by using R package “ggstatsplot” (https://github.com/IndrajeetPatil/ggstatsplot, accessed on 15 March 2022).

### 4.6. Functional Enrichment Analysis

Functional enrichment, Gene Ontology (GO), and Kyoto Encyclopedia of Genes and Genomes (KEGG) analyses were conducted via g:GOSt (https://biit.cs.ut.ee/gprofiler/gost, accessed on 29 July 2022) to explore different molecular mechanisms and pathways involved between high and low RNA-seq expressers.

### 4.7. Single-Cell RNA-Seq Data Processing

The single-cell RNA-seq data were analyzed as described previously [42], and violin plots were drawn using R.

### 4.8. Cell Lines and Transfection

Parental MCF7 breast cancer, NCI-H460 large-cell lung carcinoma, A549 human pulmonary carcinoma, HepG2, Sk-hep-1 hepatocellular carcinoma, and MCF-10A mammary gland epithelial cells were purchased from Cell Resource Center, Shanghai Academy of Biological Sciences, Chinese Academy of Sciences. HCC1937 triple-negative breast cancer, NCI-H520 lung squamous carcinoma, SNU-387 pleomorphic hepatocellular carcinoma, SNU-182 hepatocellular carcinoma, and BEAS-2B normal lung epithelial cells were purchased from Procell, Wuhan Punosai Life Technology.

MCF7 was cultured in complete DMEM medium with 0.2 mg/mL insulin. MCF-10A was cultured in MCF-10A cell-specific culture medium (CM-0525, Procell, Wuhan, China) which was purchased from Procell. NCI-H460, A549, NCI-H520, HCC1937, SNU-387, and SNU-182 were in complete RPIM-1640 medium. HepG2, Sk-hep-1, BEAS-2B, LO2, and HEK293T were cultured in complete DMEM medium. All the media were supplemented with 10% fetal bovine serum and 1% penicillin/streptomycin, and all cells were cultured at 37 °C with 5% CO2. For transient knockdown studies, UBE2C-shRNA (Fitgene, Guangzhou, China) and control shRNA (sh-NC) plasmids as well as a final concentration of 60 nM of both PLK1-siRNA and control siRNA (si-NC) (Hanheng, Shanghai, China) were transfected for 24 h using Lipofectamine™ 3000 (Thermo Fisher Scientific, Waltham, MA, USA L3000075). MCF7, NCI-H460, and HepG2 cells with a stable knockdown of UBE2C were established by transfection with a UBE2C-shRNA lentiviral vector.

### 4.9. Real-Time Quantitative Polymerase Chain Reaction (qPCR)

Real-time qPCR analysis was performed as user’s manual using the *Evo M-MLV* RT Kit with gDNA Clean for qPCR (Accurate Biology, Changsha, China, AG11728) and SYBR Green Premix *Pro Taq* HS qPCR Kit (Accurate Biology, AG11701). All samples were analyzed in triplicate, and the expressions of all detected genes were normalized relative to that of *GAPDH*, which was used as an internal loading control. Primers for qPCR are listed in Table S1.

### 4.10. Western Blotting and Antibodies

Anti-*UBE2C* (Thermo Fisher Scientific, PA5-27223), anti-β-actin (Beyotime Biotechnology, Shanghai, China, AA128), and anti-IgG (Beyotime Biotechnology, A0208, A0216) antibodies were used to detect respective protein.

### 4.11. Cell Proliferation Assay

Cell proliferation assays were performed using the Cell Counting Kit-8 (CCK-8, GLPBIO, Shanghai, China) according to the manufacturer’s instructions. Briefly, cells were seeded onto 96-well plates (5 × 10^3^ cells per well) and transfected in their logarithmic growth phase. The cells were then added to 10 μL of CCK-8 for 1 h at 37 °C on designated days. The absorbance was measured at 450 nm using microplate reader (BioTek SYNEGRY H1, Aglient, Santa Clara, CA, USA).

### 4.12. Clone Formation Experiment

Cells were seeded in 6-well plate with 3000 cells per well. After culturing for 14 days, the culture medium was discarded, the cells were washed carefully with PBS once, and 1 mL 4% paraformaldehyde solution was added to each well to fix the cells for 30 min. The 4% paraformaldehyde solution was aspirated, the cells were washed carefully with PBS twice, 1 mL crystal violet stain solution was added to each well, and it was left at room temperature for 10 min. The crystal violet was recycled, each well was washed with distilled water, and the culture plate was placed upside down on absorbent paper to absorb the water. It dried naturally and we took pictures using digital camera. We counted the number of clones with more than 10 cells under the microscope (4× magnification) or took photos under shadowless lamp. Finally, the number of clones was calculated using ImageJ version 1.43.

### 4.13. Cell Migration Assay

A wound-healing assay was performed to detect the migration of three kinds of cancer cell lines. Cells growing in the log phase were trypsinized and seeded in 24-well plates until confluent. The cell layer was transfected with si-*PLK1* and control si-NC and wounded using a sterile tip. After incubating for 0 to 72 h, cells were photographed under an inverted microscope every 24 h. The distance between the two edges of the scratch (wound width) was measured at 8 sites using ImageJ for each image (4× magnification). MCF7, NCI-H460, and HepG2 cells with a stable knockdown of *UBE2C* and control sh-NC were transiently transfected with si-*PLK1* for 48 h. Afterwards, Falcon^®^ Permeable Support for 24-well Plate with 8.0 µm Transparent PET Membrane (Corning Inc., Corning, NY, USA) was used for measuring cell migration. After taking pictures of the cells, we added 1 mL absolute ethanol per well. After sufficiently shaking them, the light absorbance was measured at 570 nm and compared to the curves of the control samples.

### 4.14. Cell Apoptosis Assay

Stable-knockdown *UBE2C* cells were treated with si-*PLK1* and control si-NC for 48 h, respectively. Afterwards, the cells were mixed with trypsin without EDTA. After termination, they were centrifuged at 1000 rpm for 5 min to remove the supernatant containing trypsin and then washed 2 times with PBS. The cells were regenerated with serum-free medium; afterwards, cell apoptosis was analyzed using Annexin V-FITC Apoptosis Detection Kit (Beyotime Biotechnology, C1062M) according to the instructions.

### 4.15. Cell Cycle Assay

Stable-knockdown *UBE2C* cells were treated with si-*PLK1* and control si-NC for 48 h, respectively. We carefully collected the culture medium into a centrifuge tube to be used in 1× propidium iodide staining solution. Afterwards, the cells were mixed with trypsin and cell resuspension was collected in the centrifuge tube with the culture medium before being centrifuged at 1000 rpm for 5 min to pelletize cells. Carefully, we removed the supernatant and resuspended the cell pellet with ice-cold PBS. We transfered the cell resuspension to a new 1.5 mL centrifuge tube, centrifuged it again, and carefully removed the supernatant. The cells were fixed with precooled 70% ethanol at 4 °C for 30 min. The cells were regenerated with ice-cold PBS; afterwards, cell cycle was analyzed using Cell Cycle and Apoptosis Analysis Kit (Beyotime Biotechnology, C1052) according to the instructions.

### 4.16. RNA-Seq

Total RNA was extracted from the cells transfected for 48 h using UNlQ-10 Column Total RNA Purification Kit (Sangon Biotech, Shanghai, China, B511361-0100) and stored at −80 °C. The complementary DNA (cDNA) libraries of each pooled RNA sample for single-end sequencing were generated using the NEBNext^®^ UltraTM RNA Library Prep Kit for Illumina^®^ (NEB, Cat. No. E7530L, New England Biolabs, Ipswich, MA, USA) according to the manufacturer’s instructions. The cDNA libraries were subjected to the NovaSeq 6000 system (Illumina), according to commercially available protocols. The changed RNAs were validated via quantitative PCR using the primers listed in Table S1.

### 4.17. RNA-Sequence Analysis

RNA-seq data quality control software fastp (v0.23.2) was first used to produce clean reads with default parameters. The clean reads were further aligned to GRCH38.p13 reference genome with Hisat2 (v2.2.1). Samtools (v1.9) was used to convert the alignment file format. The Counts function of subread (v2.0.1) was used to calculate and generate gene expression profiles. The genes were annotated using gtf file from GENCODE (v36).

### 4.18. Trend Analysis

The trend of gene abundance changes in continuously changing samples or groups was found and visualized by using online analysis tools from GENE DENOVO (https://www.omicshare.com/tools/, accessed on 14 April 2022). Briefly, the TPM value of DGEs in each group was input, and the first time point (control group, Lv-NC+si-NC) was used as the control to calculate the expression difference of all samples relative to the first time point. A fold change > 1.2 was set as the statistical threshold value of minimum change multiple. *p* < 0.05 indicated statistically significant differences. Analysis and visualization of gene quantity and *p*-value trend profiles were performed with Dynamic Trend online tool, as well as concrete data.

### 4.19. Statistical Analysis

Datasets were analyzed using Prism 9.0 software; mean ± SEM values are shown. Data were expressed as the mean values with the standard error of the mean. Statistical differences were determined using the Student’s *t*-test or one-way ANOVA and Tukey’s multiple comparison test. * *p* < 0.05; ** *p* < 0.01; *** *p* < 0.001; **** *p* < 0.0001; n.s., not significant.

## Data Availability

The data presented in this study are available on request from the corresponding author.

## Supplementary Materials

The following supporting information can be downloaded at: https://www.mdpi.com/article/10.3390/ijms242115658/s1.
