# Supplementary material for: Combined Inhibition of UBE2C and PLK1 Reduce Cell Proliferation and Arrest Cell Cycle by Affecting ACLY in Pan-Cancer
Source: Int J Mol Sci. 2023 Oct 27;24(21):15658. doi: 10.3390/ijms242115658 (PMC10650476; doi:10.3390/ijms242115658)
Supplement: Supplementary file 1 [file ijms-24-15658-s001.zip › TableS1.pdf]

Table S1. The primers used in this study.

Name	Forward sequence	Reverse sequence	Application
sh*UBE2C*	GATCTGTATGATGTCAGGACCATTCCTCGAGGAATGGTCCTGACATCATACATTTTT	AATTAAAAATGTATGATGTCAGGACCATTCCTCGAGGAATGGTCCTGACATCATACA	Interfering UBE2C
shNC	GATCTTCTCCGAACGTGTCACGTCTCGAGACGTGACACGTTCGGAGAATTTTT	AATTAAAAATTCTCCGAACGTGTCACGTCTCGAGACGTGACACGTTCGGAGAA	
si-*PLK1*	CUAAGUCUCUGCUGCUCAA	UUGAGCAGCAGAGACUUAG	Interfering *PLK1*
si-*BIRC5*	AGCCCUUUCUCAAGGACCATT	UGGUCCUUGAGAAAGGGCUTT	Interfering *BIRC5*
si-NC	UUCUCCGAACGUGUCACGUTT	ACGUGACACGUUCGGAGAATT	
FAM-NC	UUCUCCGAACGUGUCACGUTT	ACGUGACACGUUCGGAGAATT-FAM	
GAPDH	ACAACTTTGGTATCGTGGAAGG	GCCATCACGCCACAGTTTC	
UBE2C	GACCTGAGGTATAAGCTCTCGC	CAGGGCAGACCACTTTTCCTT	qPCR
PLK1	AGAAAGGGCACAGTTTCGAG	GGGTTGATGTGCTTGGGAATA	qPCR
S1P	ATACAGTTGTGGAATATGAATATATTG	ATGTTTCGAGGTATAATTCTCCAATTG	
S2P	GCTTGGTGAAGTCATCTGTCTATT	GCTTCCAGCCAGTCTTCATAAGAATGT	
ATF6	ACCACTAGTAGTATCAGCAGGAAC	AATGTGTCTCCCCTTCTGCG	
CDK2	GCATCTTTGCTGAGATGGTGAC	ACTTGGGGAAACTTGGCTTGT	
ACLY	TCGGCCAAGGCAATTTCAGAG	CGAGCATACTTGAACCGATTCT	
ACACA	ATGTCTGGCTTGCACCTAGTA	CCCCAAAGCGAGTAACAAATTCT	

FASN	AAGGACCTGTCTAGGTTTGATG C	TGGCTTCATAGGTGACTTCCA	
SCD	TCTAGCTCCTATACCACCACCA	TCGTCTCCAACTTATCTCCTCC	
SREBP1	CTCCCTAGGAAGGGCCGTA	GCCGACTTCACCTTCGATGT	
IDH1	TGCAAAAATATCCCCCGGCT	TTTGGGTTCCGTCACTTGGT	
ABCG5	GGCCACCAGAAAATTTGCCC	GTAGGAGGCATGGAGGATGC	
ABCG8	CACTCGCAGCCCTGTTTCTA	GCATCTTCGTAGGACTCGGG	
ACSS2	TTACTGGGAGGGCAATGAGC	CCCCTTCTGAATGCCCTGTT	
JAK2	TCTGGGGAGTATGTTGCAGAA	AGACATGGTTGGGTGGATACC	
STAT3	CAGCAGCTTGACACACGGTA	AAACACCAAAGTGGCATGTGA	
CDK1	CCCTTTAGCGCGGATCTACC	CATGGCTACCACTTGACCTGT	
CDK4	ATGGCTACCTCTCGATATGAGC	CATTGGGGACTCTCACACTCT	
CDK6	TGAGGTTAGAGCCATCTGGAAA	TCTTCATTCACACCGAGTAGTGC	
C-myc	GTCAAGAGGCGAACACACAAC	TTGGACGGACAGGATGTATGC	qPCR
BTG2	GGTTTCCCGAAAAGCCGTCCA	CCCAATGCGGTAGGACACCTC	
Bax	CGAGTGGCAGCTGACATGTTT	TTCTTCCAGATGGTGAGCGAG	
Bcl-2	ACGGGGTGAACTGGGGGAGGA	TGTTTGGGGCAGGCATGTTGACT T	
