# Supplementary figures and images for: Combined Inhibition of UBE2C and PLK1 Reduce Cell Proliferation and Arrest Cell Cycle by Affecting ACLY in Pan-Cancer
Source: Int J Mol Sci. 2023 Oct 27;24(21):15658. doi: 10.3390/ijms242115658 (PMC10650476; doi:10.3390/ijms242115658)

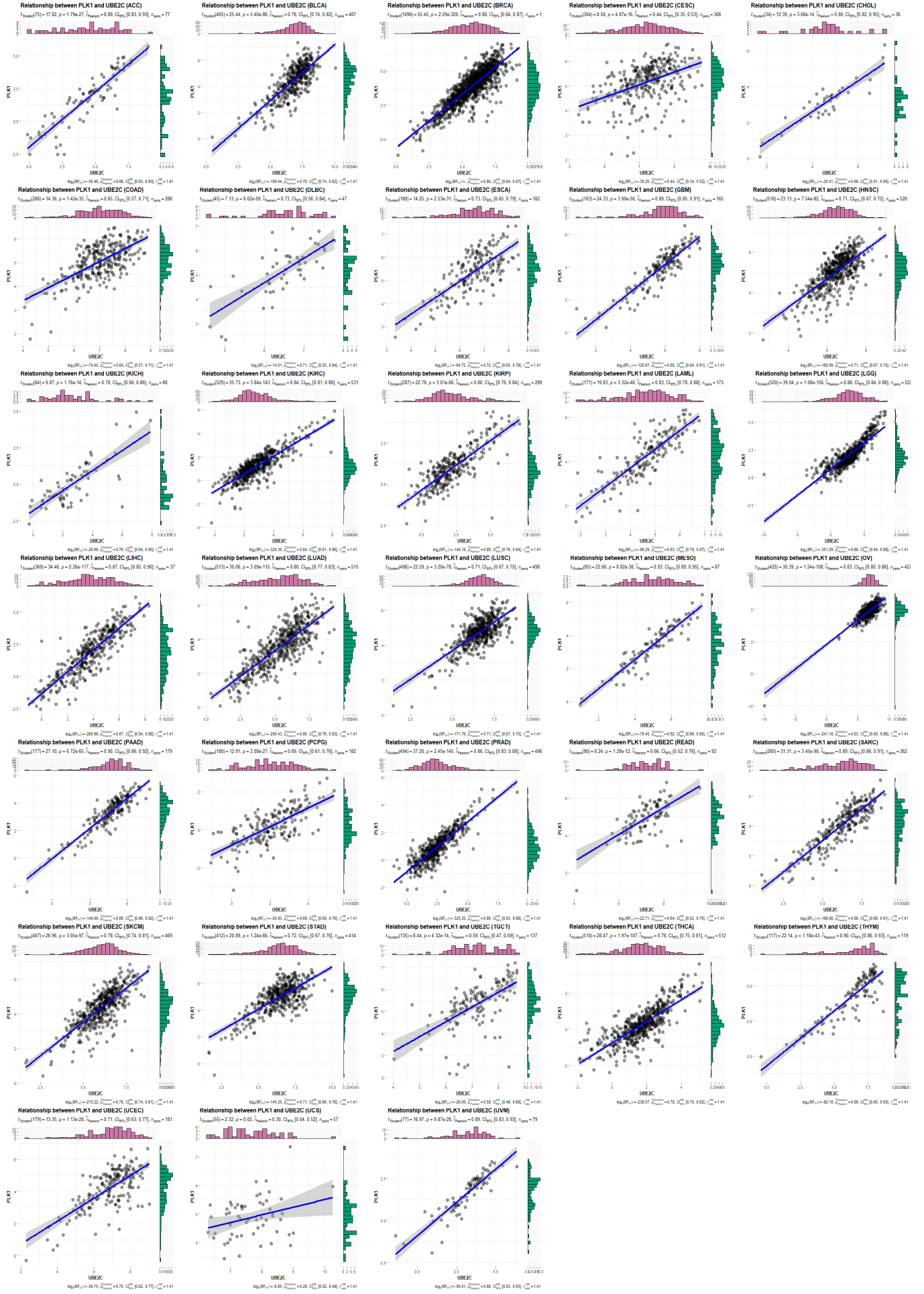

Supplement: Supplementary file 1 [file ijms-24-15658-s001.zip › FigureS2.pdf]

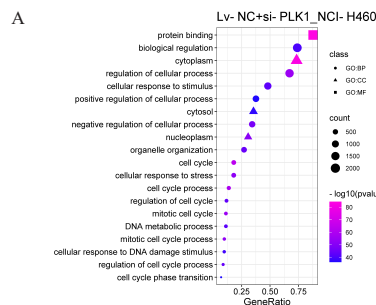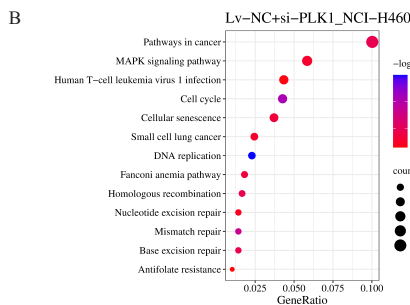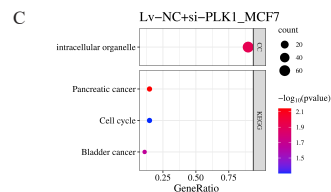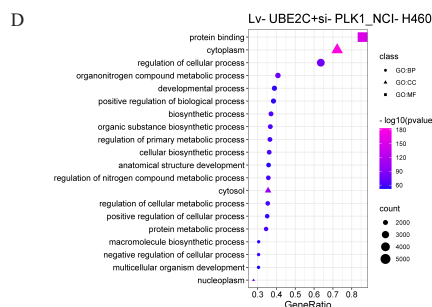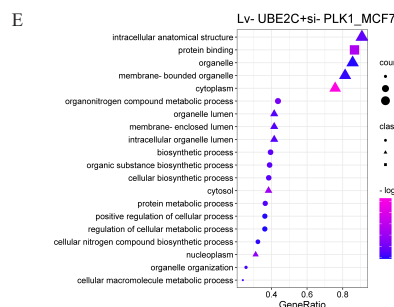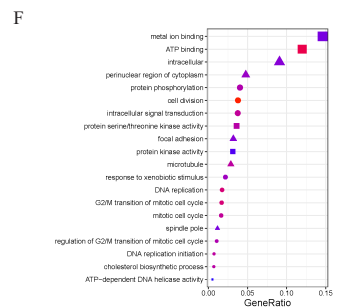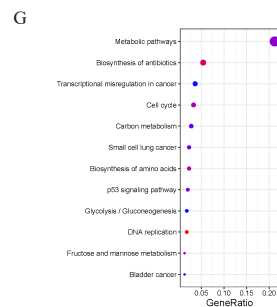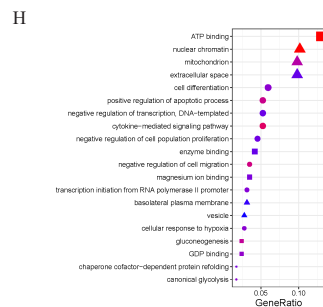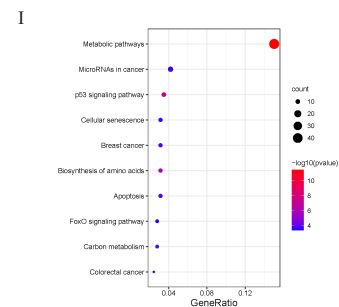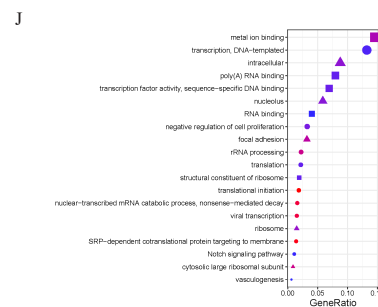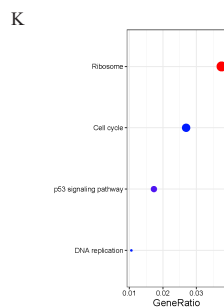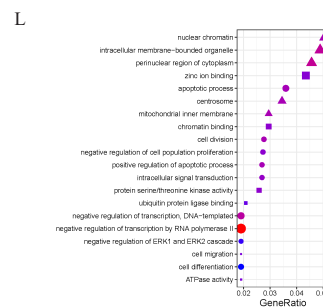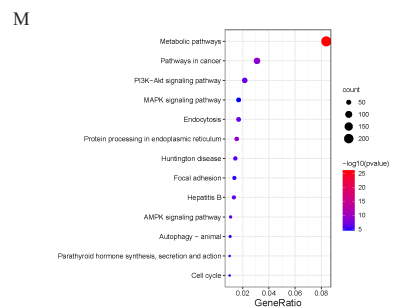

Supplement: Supplementary file 1 [file ijms-24-15658-s001.zip › FigureS3.pdf]

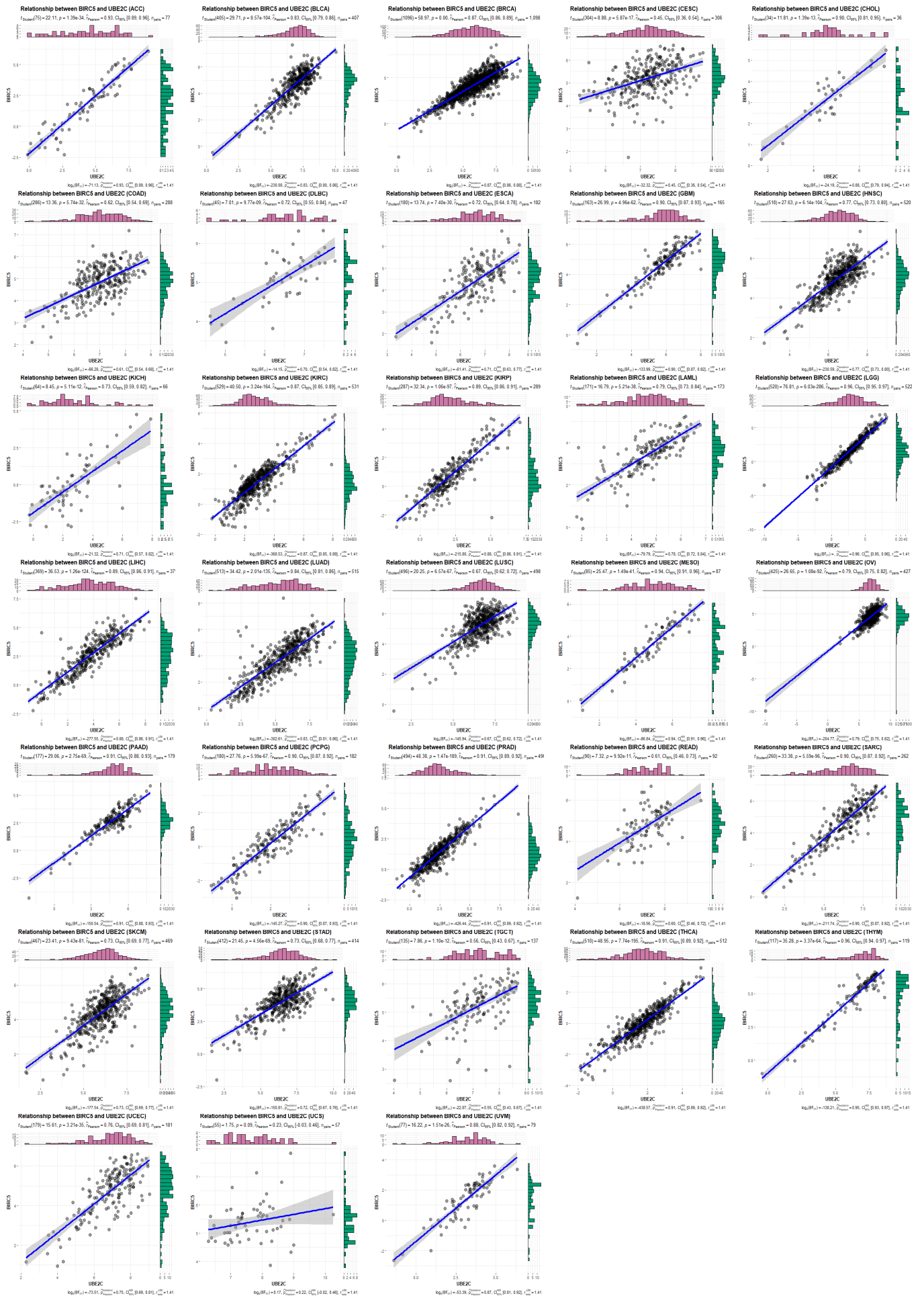

Supplement: Supplementary file 1 [file ijms-24-15658-s001.zip › FigureS4.pdf]

A

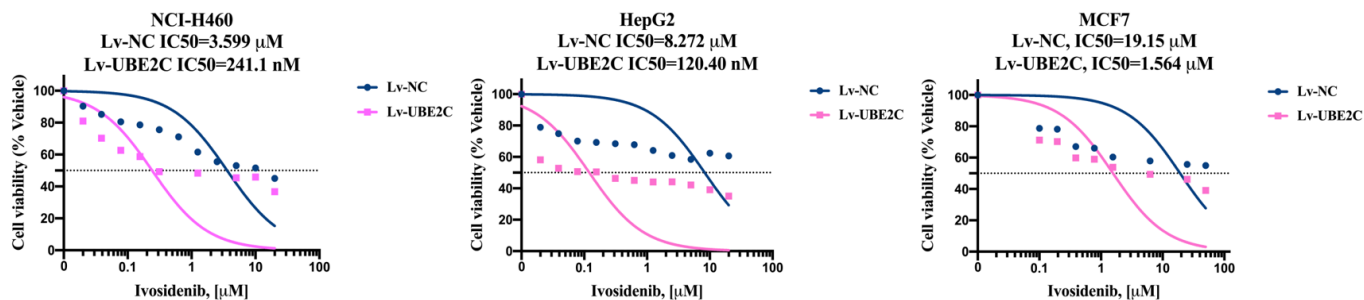

B

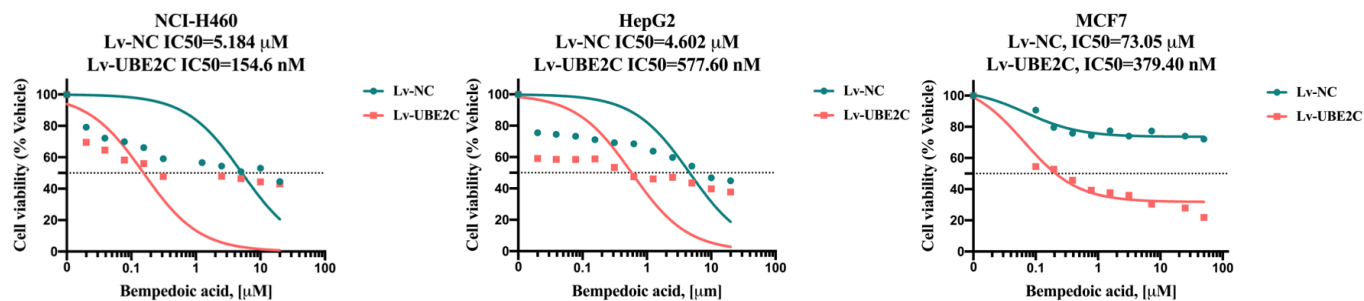

C

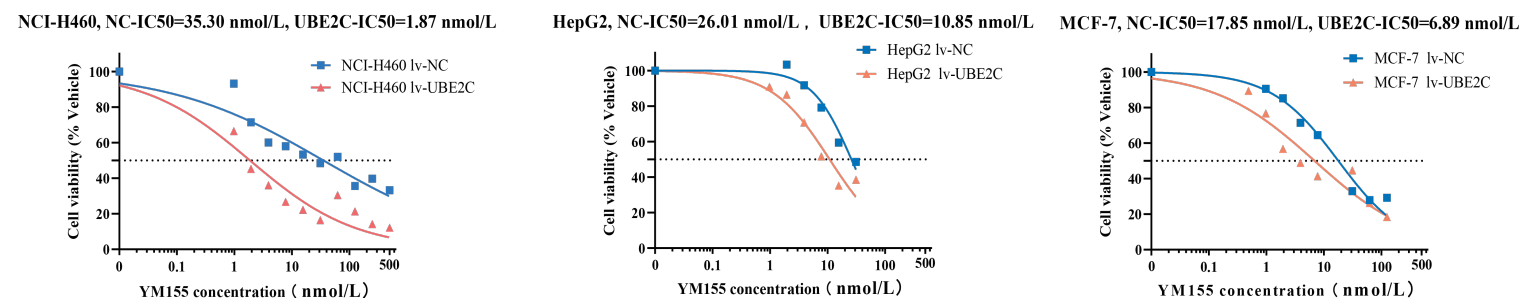

D

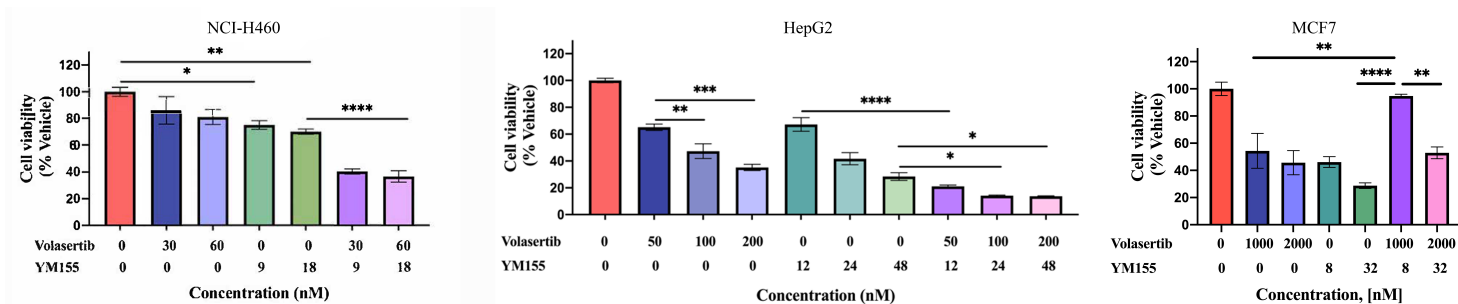

E

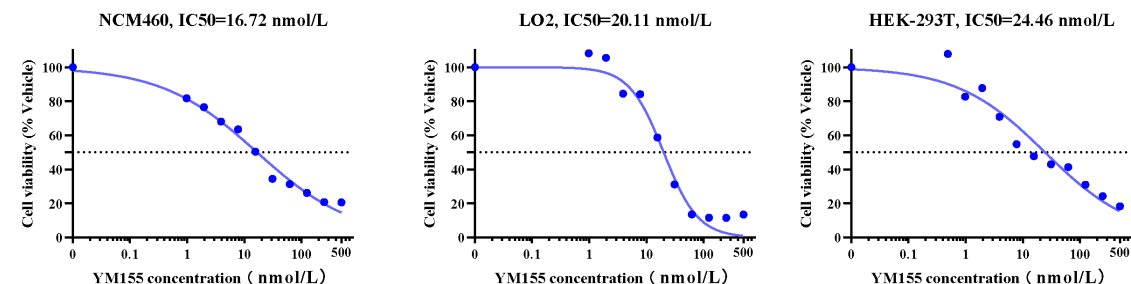

Supplement: Supplementary file 1 [file ijms-24-15658-s001.zip › FigureS5.pdf]

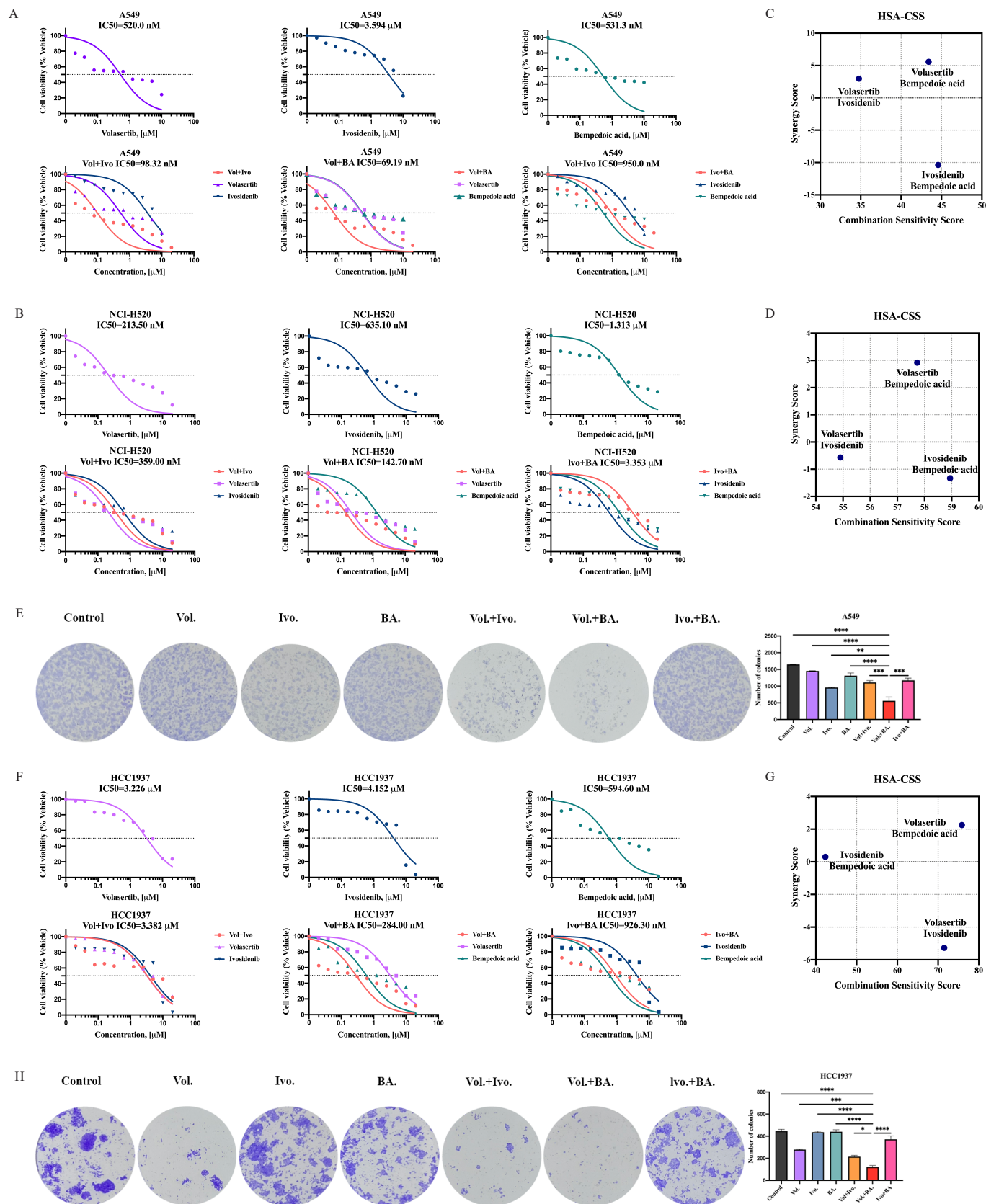

Supplement: Supplementary file 1 [file ijms-24-15658-s001.zip › FigureS6.pdf]

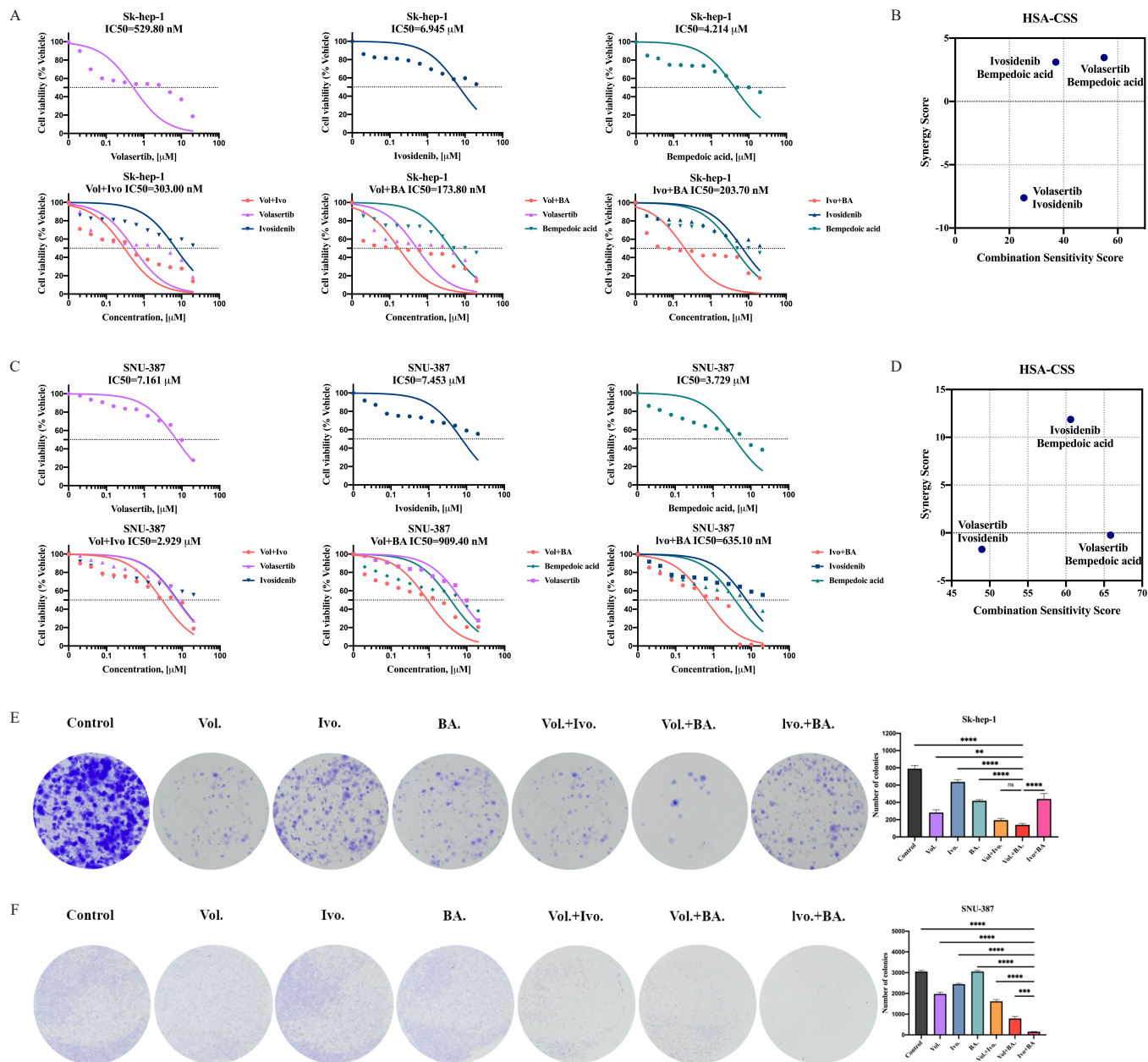

Supplement: Supplementary file 1 [file ijms-24-15658-s001.zip › FigureS7.pdf]

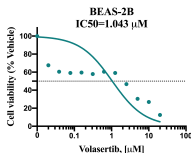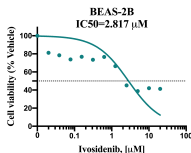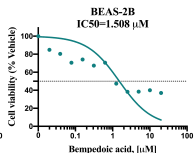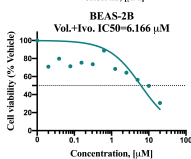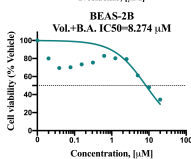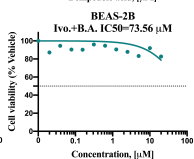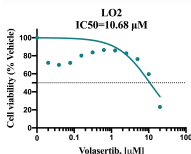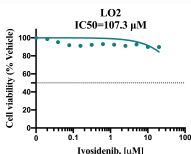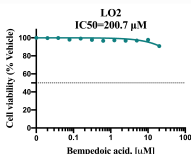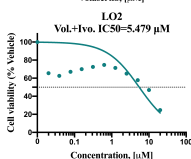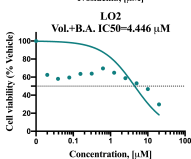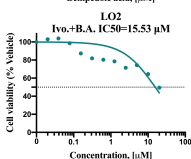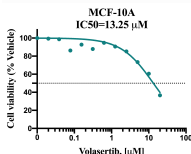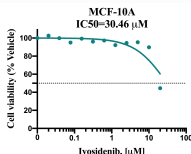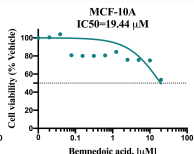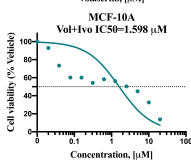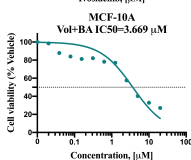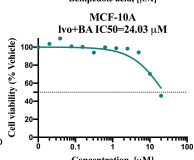

Supplement: Supplementary file 1 [file ijms-24-15658-s001.zip › FigureS8.pdf]

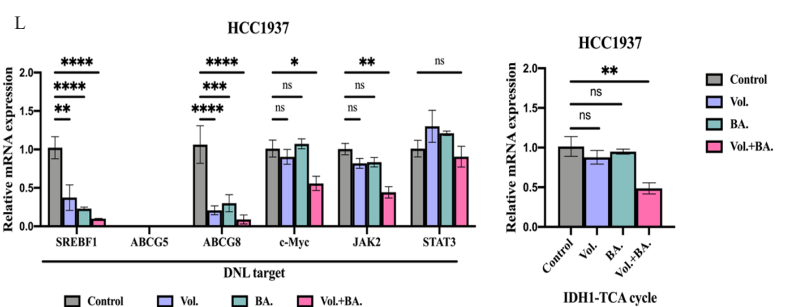

Supplement: Supplementary file 1 [file ijms-24-15658-s001.zip › FigureS9.pdf]
