# Supplementary material for: Combined Inhibition of UBE2C and PLK1 Reduce Cell Proliferation and Arrest Cell Cycle by Affecting ACLY in Pan-Cancer
Source: Int J Mol Sci. 2023 Oct 27;24(21):15658. doi: 10.3390/ijms242115658 (PMC10650476; doi:10.3390/ijms242115658)
Supplement: Supplementary file 1 [file FigureS1.pdf › FigureS1.pdf]

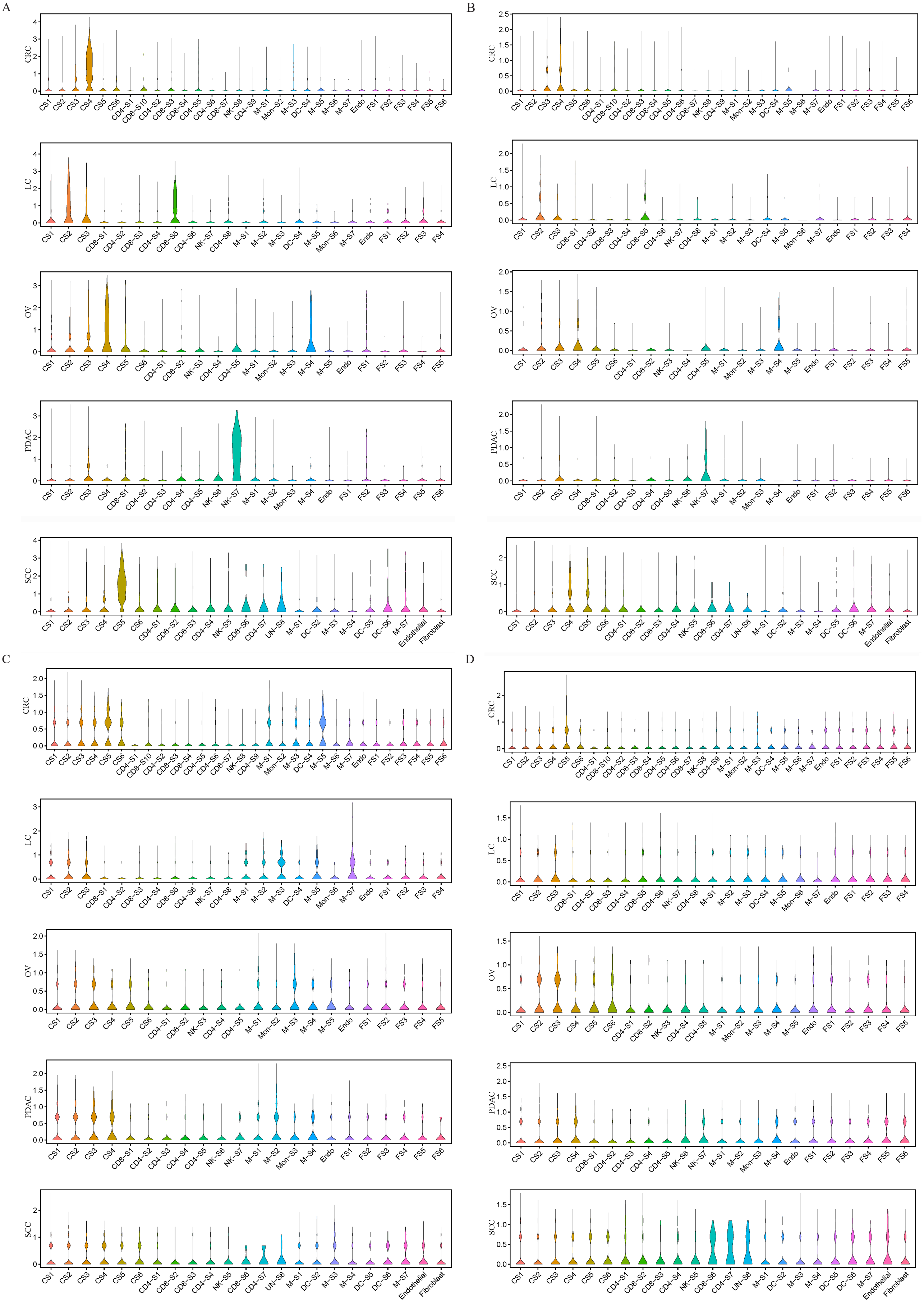
A
B
C
D
CRC
CS1 CS2 CS3 CS4 CS5 CS6 CD4-S1 CD8-S10 CD4-S2 CD8-S3 CD8-S4 CD4-S5 CD4-S6 CD8-S7 NK-S8 CD4-S9 M-S1 Mon-S2 M-S3 DC-S4 M-S5 M-S6 M-S7 Endo FS1 FS2 FS3 FS4 FS5 FS6
LC
CS1 CS2 CS3 CD8-S1 CD4-S2 CD8-S3 CD4-S4 CD8-S5 CD4-S6 NK-S7 CD4-S8 M-S1 M-S2 M-S3 DC-S4 M-S5 Mon-S6 M-S7 Endo FS1 FS2 FS3 FS4
OV
CS1 CS2 CS3 CS4 CS5 CS6 CD4-S1 CD8-S2 NK-S3 CD4-S4 CD4-S5 M-S1 Mon-S2 M-S3 M-S4 M-S5 Endo FS1 FS2 FS3 FS4 FS5
PDAC
CS1 CS2 CS3 CS4 CD8-S1 CD4-S2 CD4-S3 CD4-S4 CD4-S5 NK-S6 NK-S7 M-S1 M-S2 Mon-S3 M-S4 Endo FS1 FS2 FS3 FS4 FS5 FS6
SCC
CS1 CS2 CS3 CS4 CS5 CS6 CD4-S1 CD8-S2 CD8-S3 CD4-S4 NK-S5 CD8-S6 CD4-S7 UN-S8 M-S1 DC-S2 M-S3 M-S4 DC-S5 DC-S6 M-S7 Endothelial Fibroblast
